# Controlled Phonon Transport via Chemical Bond Stretching and Defect Engineering: The Case Study of Filled β-Mn-Type Phases

**DOI:** 10.1021/acs.inorgchem.4c02562

**Published:** 2024-09-13

**Authors:** Oleksandr Cherniushok, Taras Parashchuk, Raul Cardoso-Gil, Yuri Grin, Krzysztof T. Wojciechowski

**Affiliations:** † Thermoelectric Research Laboratory, Department of Inorganic Chemistry, Faculty of Materials Science and Ceramics, AGH University of Krakow, Mickiewicza Avenue 30, 30-059 Krakow, Poland; ‡ Max-Planck-Institut für Chemische Physik fester Stoffe, Nöthnitzer Strasse 40, 01187 Dresden, Germany

## Abstract

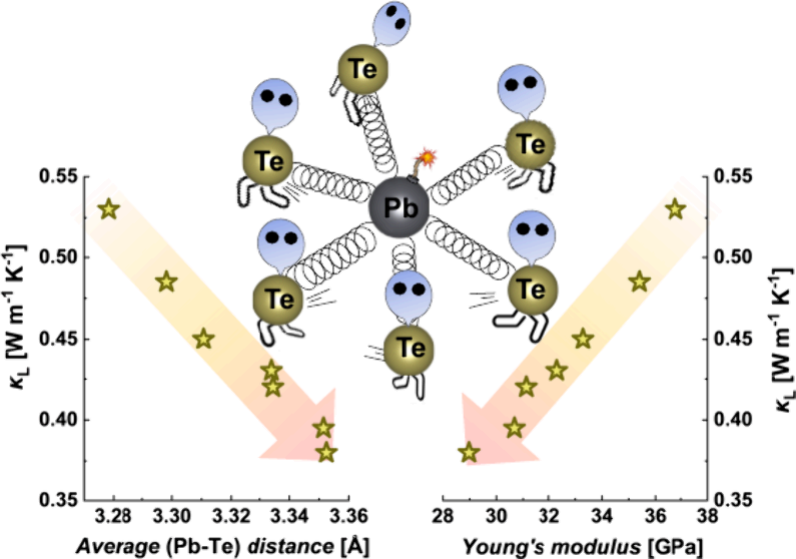

Controlling the elastic properties of the material could become a powerful tool for tuning the thermal transport in solids. Nevertheless, the impact of the crystal structure, chemical bonding, and elastic properties on the lattice thermal conductivity remains to be elucidated. This is a pivotal issue for the advancement of thermoelectric (TE) materials. In this context, the influence of cation substitution in tetrahedral voids on the structural, thermal, and TE properties of α- and β-Pb_*y*_Ga_6–*x*_In_*x*_Te_10_—filled β-Mn-type phases—is reported here. The investigated materials show semiconducting behavior and a change from p- to n-type conductivity, depending on the chemical composition and temperature. Our findings indicate that the electronic transport in β-Mn-type phases is largely influenced by the substantial distortion of the Te framework, which causes the low weighted mobility and strong scattering of charge carriers. The presence of a significant anharmonicity of lattice vibrations results in the ultralow lattice thermal conductivity of Pb_*y*_Ga_6–*x*_In_*x*_Te_10_ materials. With increasing *x*, κ_L_ decreases from 0.59 to an extremely low value of 0.36 W m^–1^ K^–1^ at 298 K due to the decrease of bonding energy, intensification of anharmonic thermal vibrations of atoms, and formation of point defects. This work demonstrates the efficacy of utilizing the crystal structure and elastic properties to regulate phonon transport in functional materials.

## Abstract 2

Phonon transport in filled β-Mn-type phases is tuned by engineering the crystal structure and elastic properties. The reduced bonding energy, enhanced anharmonic thermal vibrations of atoms, and induced point defects produce the ultralow lattice thermal conductivity of Pb_*y*_Ga_6−*x*_In_*x*_Te_10_.

## 1 Introduction

Understanding the crystal structure effect on the electronic and thermal transport properties is of essential interest for thermoelectric (TE) materials science. TE materials can be used as the main constructing elements in renewable technologies for the conversion of waste heat into electricity or for solid-state refrigeration.^1,2^ The performance of the TE materials is estimated by its dimensionless figure of merit *ZT* = *S*^2^σ*T*/(κ_e_ + κ_L_), where *S* is the Seebeck coefficient, σ is the electrical conductivity (σ = 1/ρ), *T* is the absolute temperature, and κ_e_ and κ_L_ are the electronic and lattice contributions to the thermal conductivity, respectively.^2−4^ Because *S*, σ, and κ_e_ are interconnected through the carrier concentration *n*, just a simple increase of the power factor *S*^2^σ will not be effective for the *ZT* improvement.^5,6^ The only parameter that can be optimized relatively independently is the lattice thermal conductivity κ_L_.^7^ Therefore, more attention should be paid to the TE material quality factor *B*, which is proportional to μ_w_/κ_L_, where μ_w_ is the weighted mobility of the charge carriers. The ratio of μ_w_/κ_L_ reflects the maximum attainable *ZT* for a TE material originating from its fundamental material properties (μ_w_ and κ_L_).^8^

The TE phenomenon is widely described in the literature from the thermodynamic and physical points of view.^9^ However, very few crystal structure particularities that affect the transport properties have been defined clearly until now. The establishment of the effect of the crystal structure changes on the transport properties is still challenging. Recently, many intrinsic phonon scattering mechanisms originating from the crystal structure complexity and chemical bonding hierarchy were explored and successfully implemented to reduce the lattice thermal conductivity in TE materials.^8,10−13^ Rattling atoms in the frame of the “phonon-glass and electron-crystal” (PGEC) concept,^9,14^ liquid-like partial lattices of superionic conductors described by the “phonon-liquid electron-crystal” (PLEC) concept,^7,15−17^ layered structures,^1,18,19^ lone-pair-electron-induced lattice anharmonicity,^20−24^ and theory of bonding inhomogeneity^11,25−27^ are the most intensively discussed among them. The PGEC and PLEC concepts were developed to understand the reduction of κ_L_ through the rattling-like or liquid-like behavior of “filler” atoms, keeping the high carrier mobility μ_w_ through the ordered framework of the further participating atoms in the crystal structure. As a result, these strategies should lead to a high μ_w_/κ_L_ ratio and, therefore, a high material quality factor *B*.

Recently, the TE properties of a group of ternary tellurides with a structural organization similar to that of the β-Mn-type structure were explored.^28−30^ These filled β-Mn-type phases have the chemical composition *M*_2/*n*_^*n*+^*Tr*_6_^3+^*Q*_10_^2–^ (*M*^*n*+^ = Li^+^, Na^+^, Ag^+^, Ca^2+^, Sn^2+^, Pb^2+^, and Yb^2+^; *Tr*^3+^ = Al^3+^, Ga^3+^, and In^3+^; *Q*^2–^ = Se^2–^ and Te^2–^) and are characterized by close packings of *Q*_4_ tetrahedra (similar to the arrangement of manganese atoms in cubic β-Mn). The *M*^+^ ions fill all available distorted octahedral or metaprismatic voids, while the *M*^2+^ ions occupy only half of them and *Tr*^3+^ ions are distributed in an ordered way over only 15% of the tetrahedral voids.

The investigation of the Pb–Ga–Te system has shown that the Pb_*y*_Ga_6_Te_10_ phase exists within the homogeneity range of *y* = 0.9–1.1.^31^ The deviation from the stoichiometric composition of PbGa_6_Te_10_ leads to a tuning of its charge transport and can be used for the effective modification of the Seebeck coefficient and electrical conductivity. In turn, deviation from stoichiometry induces the formation of structural defects, which effectively scatter phonons and lead to the reduction of κ_L_ in comparison with the stoichiometric material. A further study of homologous *M*_2/*n*_^*n*+^Ga_6_Te_10_ (*M* = Pb, Sn, Ca, Na, and Na+Ag) materials, considering the substitution of cations in distorted octahedral voids,^22^ has shown that the strongly disturbed thermal transport observed in the filled β-Mn-type phases originates from a three-dimensional Te–Ga network with lone-pair-like interactions on Te. This phenomenon leads to large variations of Ga–Te and *M*–Te bonding, inducing lattice anharmonicity. Moreover, the mixed-cation compound NaAgGa_6_Te_10_ shows ultralow thermal conductivity (∼0.25 W m^–1^ K^–1^ at 298 K) due to the synergistic effect of the cation disorder and bonding inhomogeneity. Although the bonding analysis gives essential information about the origins of low lattice thermal conductivity in *M*_2/*n*_^*n*+^Ga_6_Te_10_, the performed investigations did not discuss the effect of cation substitution in tetrahedral voids, which can strongly affect the electronic and thermal transport.

In this work, we performed a detailed study of the crystal structure and TE properties of α- and β-Pb_*y*_Ga_6–*x*_In_*x*_Te_10_ (*x* = 0–6 and *y* = 1)—filled β-Mn-type phases—considering the substitution of cations in tetrahedral voids. Even if the crystal structure of ordered α-PbGa_6_Te_10_ is already discussed in the literature, the crystal structure of α-PbIn_6_Te_10_ and possible structural phase transitions in the Pb_*y*_Ga_6–*x*_In_*x*_Te_10_ system are proposed for the first time. The suggested analysis of the crystal structure also clarifies the origins of electronic transport in the filled β-Mn-type phases and broadens our understanding of their ultralow lattice thermal conductivity.

## 2 Experimental Details

### 2.1 Preparation

The synthesis of all samples in the system PbTe–Ga_2_Te_3_–In_2_Te_3_ (Figure S1) with composition Pb_*y*_Ga_6–*x*_In_*x*_Te_10_ (*x* = 0–6 and *y* = 1) was carried out in graphite-coated (to avoid possible reaction with SiO_2_) quartz ampules, evacuated to a residual pressure of 10^–5^ mbar, and sealed with an oxygen-gas burner flame. The ampules were washed in the 1:3 HNO_3_/HCl concentrated acid mixture, thoroughly cleaned with distilled water and isopropyl alcohol, and finally dried. Polycrystalline samples were synthesized in a muffle furnace by reacting the elements Pb (Alfa Aesar, 99.999%), Te (Alfa Aesar, 99.999%), In (Alfa Aesar, 99.999%), and Ga (Alfa Aesar, 99.9999%) at 1223 K during 5 h. Then, the furnace was cooled in the inertial mode to room temperature. For homogenization of the materials, the resultant dark-gray and brittle ingots were ground to powder, cold-pressed, resealed, and annealed for 200 h at 823 K in evacuated quartz ampules. After the annealing process, the samples were cooled with a furnace to room temperature.

Resultant polycrystalline materials were manually milled into fine powders in an agate mortar and, prior to consolidation, characterized by powder X-ray diffraction (XRD). The powders were densified by the spark plasma sintering (SPS) technique at 773 K for 20 min in a 10-mm-diameter graphite mold under an axial pressure of 50 MPa in an argon atmosphere. The heating and cooling rates were 50 and 20 K/min, respectively. The obtained samples with a diameter of 10 mm and a length of approximately 12 mm have more than 98% of the theoretical density, determined by the Archimedes method. From the initial cylinder, a disk with a thickness of 2 mm was cut and polished for thermal transport measurements. The remaining part of the samples was cut into bars of 10 × 10 × 2 mm and used for electronic transport measurements (Figure S2).

### 2.2 Structural and Thermal Analysis

Phase identification was performed with a Bruker D8 Advance X-ray diffractometer using Cu Kα radiation (λ = 1.54185 Å, Δ2θ = 0.005°, and 2θ range 10–120°) with Bragg–Brentano geometry. Rietveld refinement of the crystal structure was performed using the *WinCSD* program package.^32^

Thermal analysis was performed on a differential scanning calorimetry (DSC) equipment (Netzsch DSC 404 C and DSC 404 F3 Pegasus) using a sample mass of 10–20 mg in sealed SiO_2_ ampules or aluminum crucibles covered by a lid using a heating rate of 10 K/min in argon or helium flow.

For microstructural analysis using optical and scanning electron microscopy, the samples were embedded in conductive resin and subsequently polished using 0.1 μm diamond powder in a slurry. The chemical composition was characterized by energy-dispersive X-ray absorption spectroscopy (EDXS; Philips SX-30 scanning electron microscope, standardless method) performed on the metallographic cross section of samples and wavelength-dispersive X-ray spectroscopy (WDXS) for quantitative analysis of the ternary phases, which was determined as an average of 10 points collected in the regions of the phase existence.

### 2.3 Electrical and Thermal Transport Properties

The Seebeck coefficient *S* and electrical resistivity ρ were measured by the commercial apparatus Netzsch SBA 458 Nemesis. Measurements were performed in argon flow at the temperature range of 298–773 K. The thermal diffusivity α_D_ was measured using a Netzsch LFA 457 equipment, and the specific heat capacity *C*_p_ was estimated from the Dulong–Petit limit. The samples were first spray-coated with a thin layer of graphite to minimize errors from the emissivity of the material and laser beam reflection caused by a shiny pellet surface. The thermal conductivity was calculated using the equation κ = *dC*_p_α_D_, where *d* is the density obtained by the Archimedes principle at the disks from SPS. The uncertainties of the Seebeck coefficient and electrical resistivity measurements were 7 and 5%, respectively; the uncertainty of the thermal diffusivity measurements was 3%. Although the sintered samples did not show any preferred orientation and were isotropic (Figure S3b), the TE properties were measured along the pressing direction from the same polycrystalline sintered cylinder, which was cut for different measurements as described above and is shown in Figure S2. The Hall effect was investigated by applying the four-probe method in constant electric and magnetic fields (*H* = 0.9 T) and a current through a sample of 10 mA. The speed of sound was measured at *T* = 298 K using an Olympus Epoch 650 ultrasonic flaw detector.

## 3 Results and Discussion

### 3.1 Thermal and Microstructural Analyses

Studying the phase equilibria in the Pb–Ga–Te system near the phase Pb_*y*_Ga_6_Te_10_, we have found that, depending on the composition, the ternary phase has a structural phase transition at 658–693 K.^31^ Below 658 K, the trigonal α-PbGa_6_Te_10_ modification exists (space groups *P*3_1_21 or *P*3_2_21), and above 693 K, the rhombohedral β-PbGa_6_Te_10_ (space group *R*32). Moreover, the Pb content in these phases varies according to Pb_*y*_Ga_6_Te_10_.^31^

**Figure 1:**
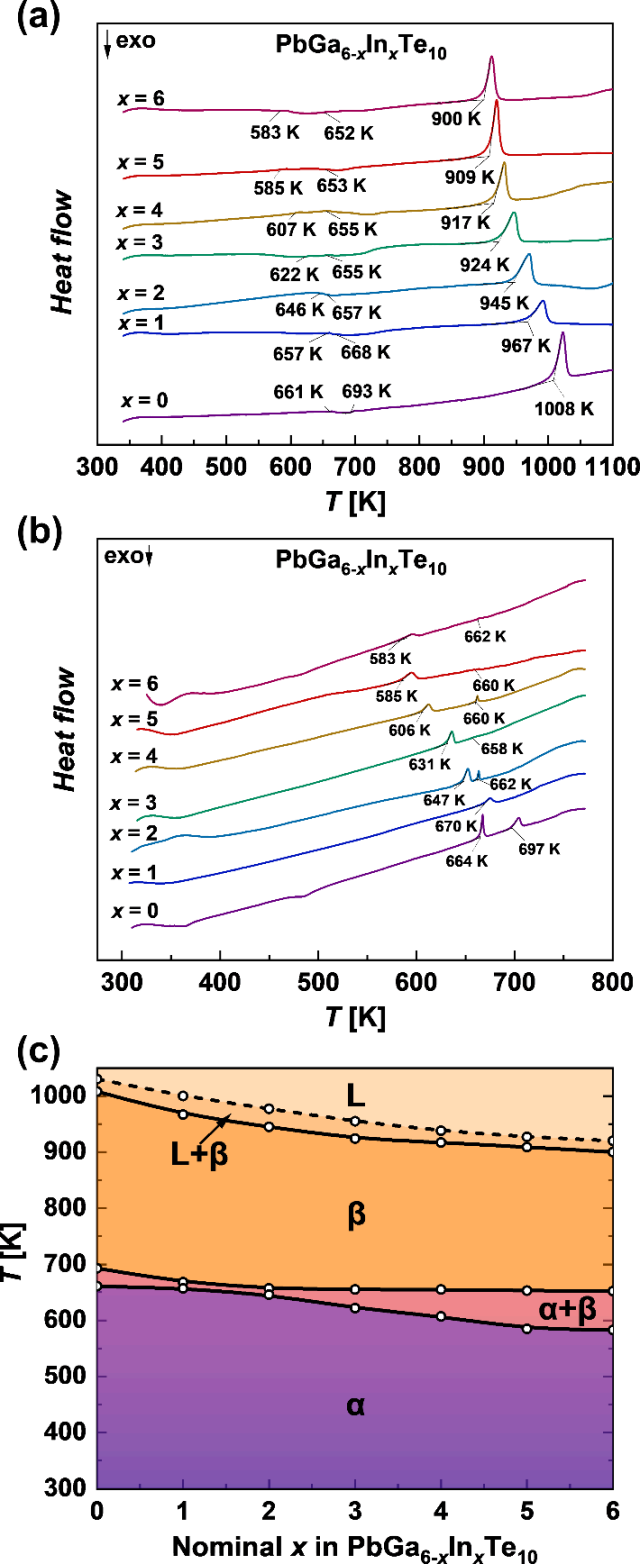
(a) Thermal behavior of Pb_*y*_Ga_6–*x*_In_*x*_Te_10_ samples (sealed quartz ampules, *T*_max_ = 1100 K, and heating rate = 10 K/min) with marked liquidus, peritectic reaction, and polymorphic phase transition temperatures, (b) samples measured in aluminum crucibles with lid (*T*_max_ = 773 K and heating rate = 10 K/min) with marked effects of polymorphic phase transitions, and (c) constructed phase diagram.

DSC analysis of Pb_*y*_Ga_6–*x*_In_*x*_Te_10_ reveals four thermal effects for all values of *x* (Figure 1a). All investigated samples show weak endothermic peaks in the range 580–700 K, reflecting the α ↔ β phase transition. Because the effects of structural phase transitions were weak and hardly visible on the DSC results using the sealed quartz ampules, they were additionally confirmed via DSC measurements of all samples in aluminum crucibles under helium flow (Figure 1b). The use of aluminum crucibles provides a higher sensitivity of the DSC signal over the phase transition range. The PbGa_6_Te_10_ sample shows a structural phase transition between 661 K (decomposition temperature of the α phase) and 693 K (formation temperature of the β phase), which is in good agreement with our previous work.^31^ With an increasing In content in Pb_*y*_Ga_6–*x*_In_*x*_Te_10_, the temperature of this phase transition decreases to 583–652 K for PbIn_6_Te_10_. All samples show broad endothermal peaks around 1000 K, which can be decomposed in at least two (Figure 1a), and correspond to incongruent decomposition of Pb_*y*_Ga_6–*x*_In_*x*_Te_10_ materials, as was already reported for PbGa_6_Te_10_.^31^ To mark these overlapping effects on the phase diagram, we chose the temperatures of the beginning and end of the endothermic peaks mentioned above. A peritectic decomposition of Pb_*y*_Ga_6–*x*_In_*x*_Te_10_ materials was additionally confirmed via powder XRD analysis of the samples after DSC measurements, which revealed mostly PbTe and (Ga,In)_2_Te_3_ (Figure S3a).

Based on the DSC analysis performed, the PbGa_6_Te_10_–PbIn_6_Te_10_ phase diagram was constructed (Figure 1c). The nonmonotonic behavior of the decomposition temperature of the α phase and the formation temperature of the β phase is most probably caused by losses of Pb during the preparation steps; i.e., the *y* value in Pb_*y*_Ga_6–*x*_In_*x*_Te_10_ is not exactly 1. For the first time, a structural phase transition is observed in PbIn_6_Te_10_. This means that in an earlier work^28^ the crystal structure of the high-temperature modification β-PbIn_6_Te_10_ with rhombohedral *R*32 symmetry was reported. This was also proven by powder XRD analysis of the samples after different thermal treatments (Figures S3–S5).

**Figure 2:**
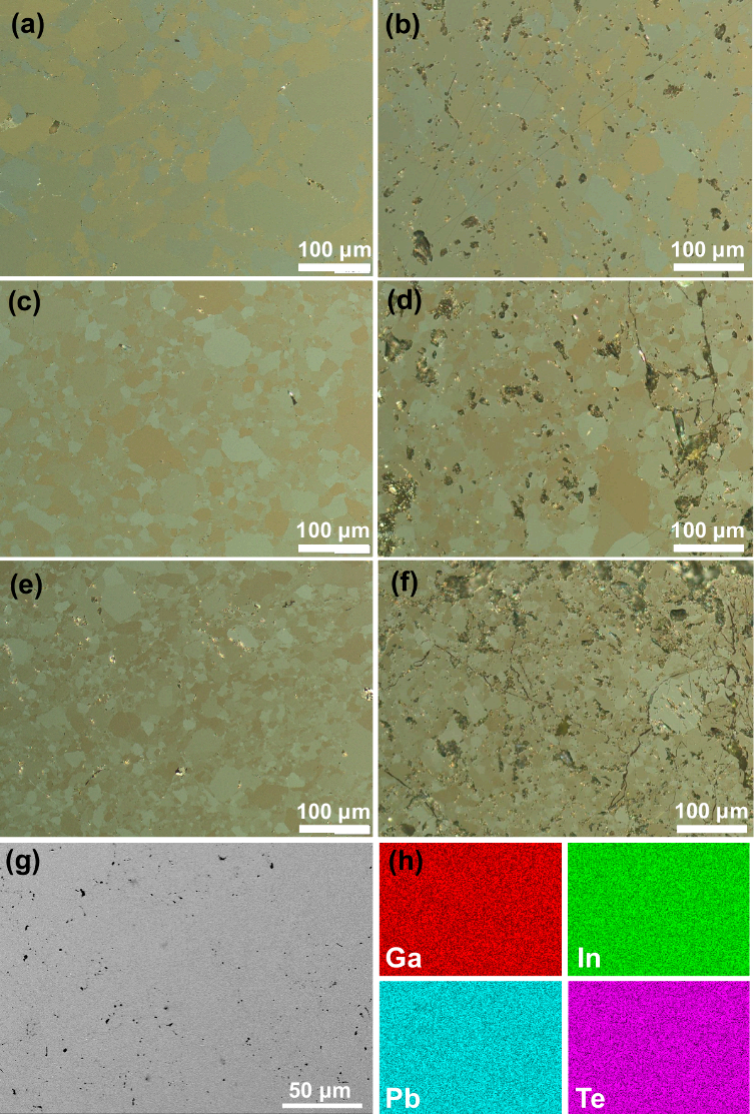
Optical polarized-light micrographs of α-Pb_*y*_Ga_6–*x*_In_*x*_Te_10_ samples after SPS treatment: (a) *x* = 0; (c) *x* = 3 (;e) *x* = 6. Optical polarized-light micrographs of α-Pb_*y*_Ga_6–*x*_In_*x*_Te_10_ samples after TE properties measurements up to 773 K: (b) *x* = 0; (d) *x* = 3; (f) *x* = 6. (g) Backscattered electron images of the α-Pb_*y*_Ga_6–*x*_In_*x*_Te_10_ sample with *x* = 3 after SPS treatment and (h) EDXS element mapping.

The microstructure of the Pb_*y*_Ga_6–*x*_In_*x*_Te_10_ samples after the SPS treatment (Figures 2a,c,e and S6) reveals their single-phase polycrystalline nature and low amount of pores, in agreement with the high densification reached (>98% of theoretic density). The regions with different colors correspond to different crystallographic orientations of the grains. The microstructure of the Pb_*y*_Ga_6–*x*_In_*x*_Te_10_ samples after TE properties measurements up to 773 K (Figures 2b,d,f and S7) shows significantly ruined grain boundaries, which is additional proof of the structural phase transition of materials in this temperature region.

Figure S8 shows backscattered electron images of Pb_*y*_Ga_6–*x*_In_*x*_Te_10_ samples after the SPS procedure with the labeled chemical composition of the phases. The obtained WDXS compositions (Table 1) show good agreement with the nominal composition. Nevertheless, slight deviations from the stoichiometry were observed in all samples. Particularly, a deficiency of Te and Pb, as well as an excess of Ga and In, was found for the majority of the samples. The element mapping of the PbGa_6–*x*_In_*x*_Te_10_ sample with *x* = 3 (Figure 2g,h) confirmed the homogeneous distribution of the components in the sample within the sensitivity of the EDXS technique.

**Table 1:**
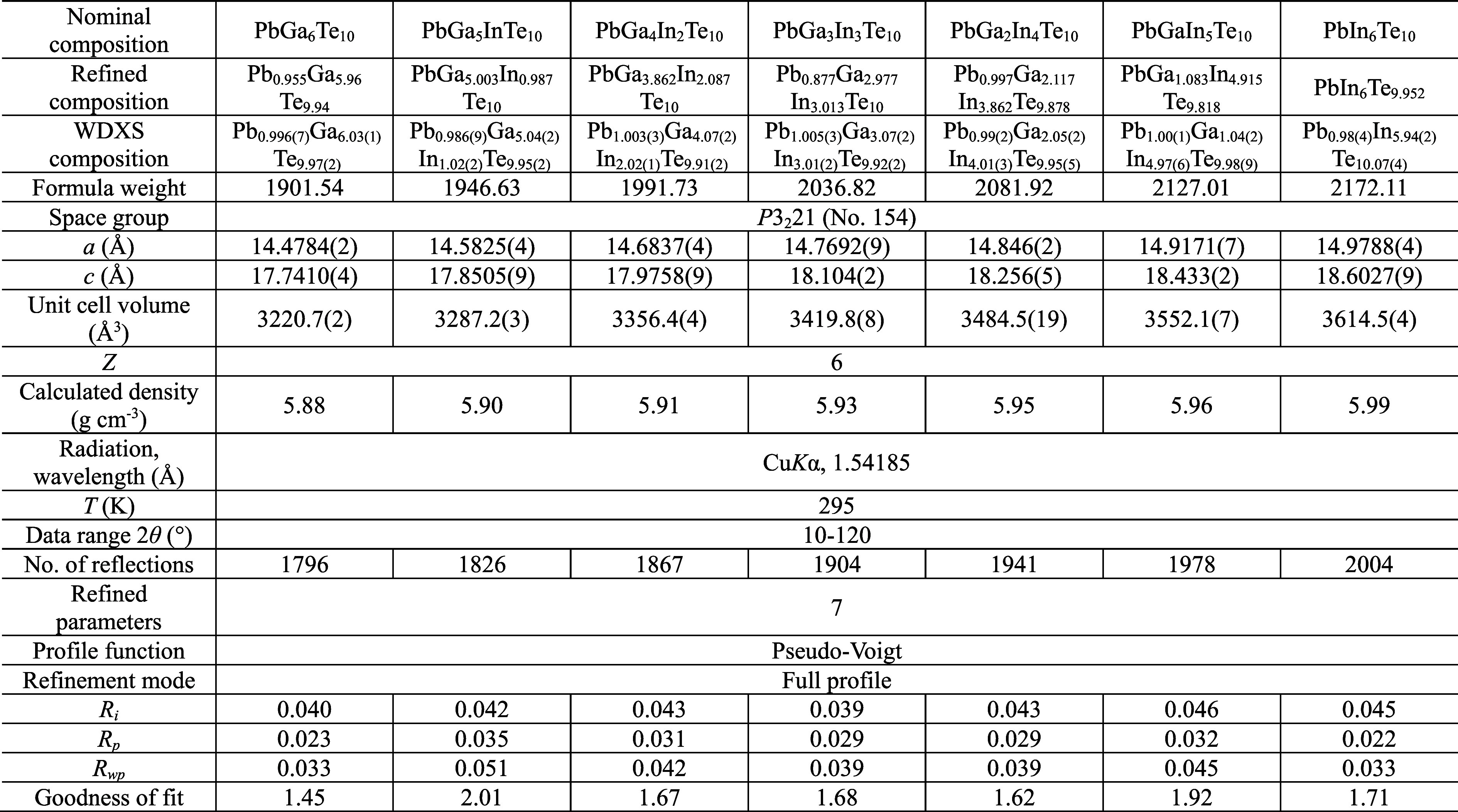
Crystallographic Information of α-Pb_*y*_Ga_6–*x*_In_*x*_Te_10_ Samples

### 3.2 Powder XRD Analysis

**Figure 3:**
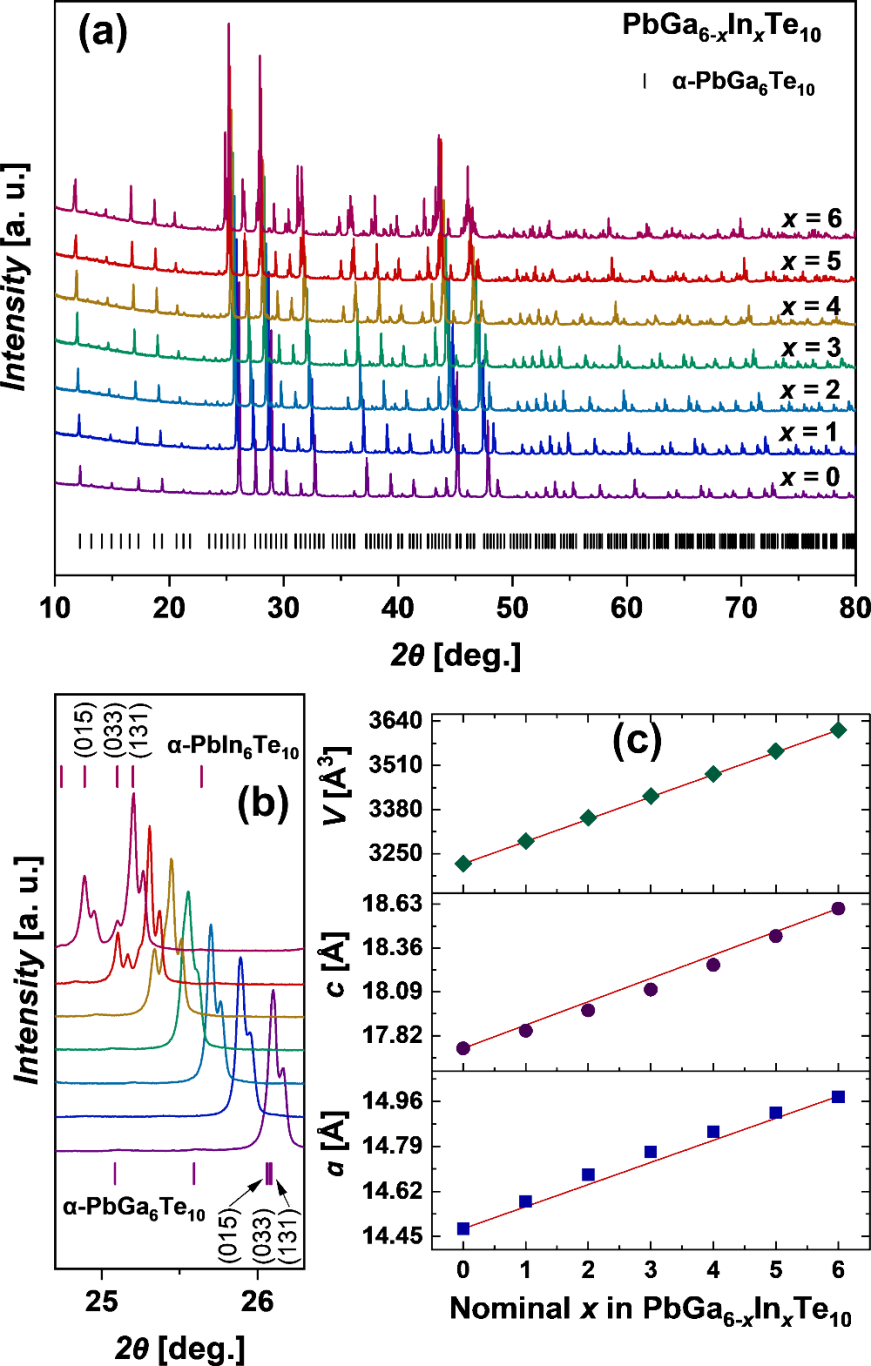
(a) Powder XRD patterns (Cu Kα radiation) of α-Pb_*y*_Ga_6–*x*_In_*x*_Te_10_ samples slowly cooled after annealing at 823 K with (b) magnified 2θ range indicating the splitting of the reflections (015), (033), and (131); (c) refined lattice parameters.

Structural analysis of synthesized Pb_*y*_Ga_6–*x*_In_*x*_Te_10_ samples after annealing was performed by using powder XRD (Figure 3a). The shift of the reflections’ positions to lower 2θ values with increasing *x* content (Figure 3b) indicates lattice expansion due to the higher covalent radius of In (1.50 Å) in comparison to Ga (1.25 Å).^33^ All of the registered reflections were successfully indexed in the space group *P*3_2_21, indicating the existence of α modification at room temperature. No additional reflections were observed. The lattice parameters of Pb_*y*_Ga_6–*x*_In_*x*_Te_10_ samples after annealing were accurately determined by least-squares refinement (Figure 3c). They showed a positive deviation from linearity for the *a* lattice parameter but a negative deviation for the *c* parameter, whereas the unit cell volume increased linearly with increasing *x*, in agreement with Vegard’s rule.^34^

**Figure 4:**
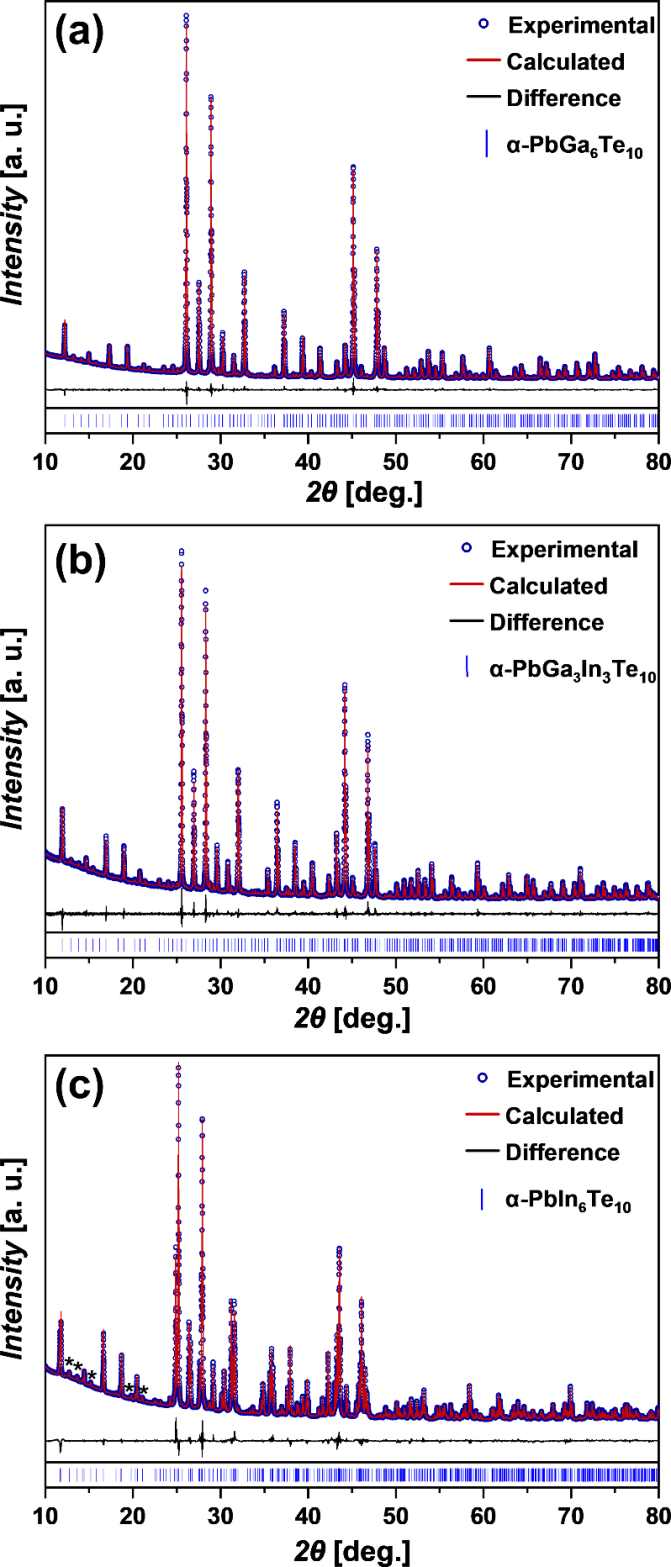
Powder XRD patterns of the PbGa_6_Te_10_ (a), PbGa_3_In_3_Te_10_ (b), and PbIn_6_Te_10_ (c) samples.

**Figure 5:**
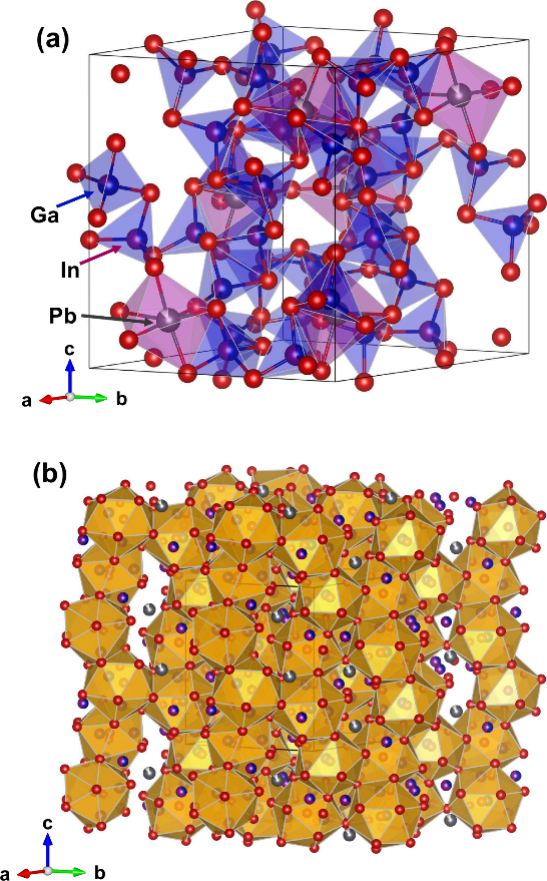
(a) Crystal structure of α-PbGa_3_In_3_Te_10_ with representative polyhedra. (b) Crystal structure representation as a packing of [(Ga,In)_3_Te_13_] icosahedra.

To understand the influence of In substitution on the crystal structure of Pb_*y*_Ga_6–*x*_In_*x*_Te_10_, we performed Rietveld structure refinement (Figure 4). Crystallographic information and final refined parameters are presented in Tables 1 and S1–S3. For refinement of the crystal structure, we used the model with primitive trigonal symmetry.^29^ All reflections of the powder XRD of PbGa_6_Te_10_ were successfully indexed in the *P*3_2_21 space group and acceptable residual values, atomic coordinates, and displacement parameters were achieved during crystal structure refinement (Table S1). Indexing the PbIn_6_Te_10_ powder pattern in the rhombohedral *R*32 space group,^28^ we noticed weak superstructure reflections (Figure 4c, asterisks), which cannot be assigned in the space group *R*32. Hence, all reflections of the powder XRD of PbIn_6_Te_10_ were also indexed in the space group *P*3_2_21 but with larger lattice parameters. The acceptable residual values, atomic coordinates, and displacement parameters were obtained using the crystal structure model of α-PbGa_6_Te_10_ (Table S2).^29^ The crystal structure refinement of all Pb_*y*_Ga_6–*x*_In_*x*_Te_10_ polycrystalline samples was also carried out using the model in the space group *P*3_2_21 (Table S3).^29^ During the refinement, it was observed that the Ga3 and Ga6 positions tend to contain more Ga, Ga4, and Ga5 positions; more In, Ga1, and Ga2 positions seem to be close to equally occupied by In and Ga. The enlarged atomic displacement parameter for some Te is most probably connected to their anisotropic character caused by the presence of larger In atoms in the coordination sphere. However, all of these are only tendencies and cannot be really quantified. More reliable is the essential defect occupation of the Pb position (Figure 5). The increase of the In content in Pb_*y*_Ga_6–*x*_In_*x*_Te_10_ samples leads to the expansion of the [(Ga,In)_3_Te_13_] icosahedra and simultaneous growth of the Pb–Te interatomic distance from 3.278 to 3.353 Å (Figure S9).

Table 1 shows the nominal and refined chemical compositions of the Pb_*y*_Ga_6–*x*_In_*x*_Te_10_ samples in comparison with the WDXS results. Most of the refined chemical compositions of Pb_*y*_Ga_6–*x*_In_*x*_Te_10_ samples show defects on the Pb and Te sites, which can be connected with some loss of these elements during sample preparation. A slight difference between the refined and WDXS chemical compositions can also be connected with some loss of these elements during measurements. Particularly, the powders after annealing were used for XRD analysis, while the WDXS experiment was done using the pieces of samples after SPS.

### 3.3 Electronic Transport

**Figure 6:**
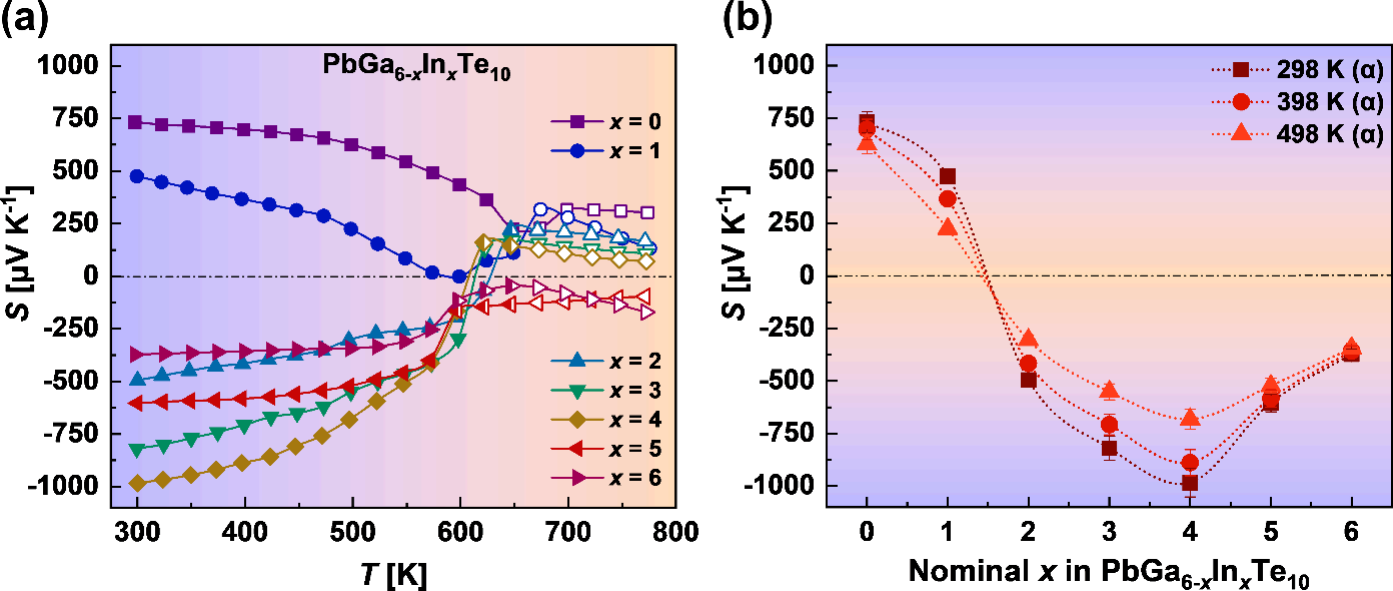
Seebeck coefficient of Pb_*y*_Ga_6–*x*_In_*x*_Te_10_ samples (a) as a function of the temperature and (b) as a function of *x* at selected temperatures. Filled symbols correspond to the temperature range of existence of the α modification and open symbols to the β modification.

The analysis of the electronic transport of the filled β-Mn-type phases is rather a complex task due to the relatively low carrier concentration (the Hall experiment is often not possible to perform due to the too-high resistivity of samples). In such a case, even a slight change in the chemical composition may cause a significant modification of the Seebeck coefficient.^35^ Moreover, the participation of p- and n-type carriers in electrical transport (caused by the simultaneous presence of Pb and Te vacancies, respectively) results in the self-compensation effect.^36−39^ Considering that the sign of the Seebeck coefficient determines the majority carriers (in n-type semiconductors, the majority carriers are electrons; in p-type semiconductors, they are holes), we can proceed with the analysis of the *S*(*T*) and *S*(*x*) dependences in Figure 6a,b. The decrease in *S* from 730 to 475 μV K^–1^ for samples with *x* = 0 and 1 is observed at room temperature. This can be explained by the increase in the concentration of electrons due to the increase in the Te deficiency (Table 1). Most probably, the compensation of holes by electrons and a change of the majority carriers occur, reflected in the sign change of *S* to negative. Hence, for samples with *x* = 2, 3, and 4, the Seebeck coefficients increase in absolute value to −500, −820, and −980 μV K^–1^, respectively. Then, the carrier concentration of the majority carriers (electrons) becomes higher, and a decrease in the absolute value of the Seebeck coefficient to −600 and −370 μV K^–1^ is observed. The schematic representation of the relationship of *S* and the electrical conductivity is shown in Figure S10.

In turn, such variations in the Seebeck coefficient may be explained by the changes in the formation of dominating defects (eqs 1–3). As was established in our previous work,^31^ the presence of intrinsic Pb vacancies (□^Pb^) in the Pb_*y*_Ga_6_Te_10_ (*y* < 1 means the □^Pb^) structure leads to the formation of holes h^+^ (eq 1) and explains the p-type behavior of this material. With In substitution on Ga sites in the PbGa_6_Te_10_-type structure, the above-mentioned increase of the Pb–Te interatomic distance can facilitate the formation of Te vacancies (□^Te^), giving rise to the electron concentration e^–^ (eq 2). Consequently, the change from p- to n-type conductivity observed in the investigated series of samples can be explained by the presence of both types of defects, Pb and Te vacancies (Figure S10). Taking into account the lowering of the melting temperature from the Ga compound to In (from around 1000 to 900 K, respectively), the loss of Te is highly probable during SPS at 773 K. Moreover, there is no other way to explain the n-type behavior of our samples with Pb defects alone because we have no excess of Pb according to the chemical analysis. This also means that the introduction of point defects on Te sites can effectively change the carrier concentration.
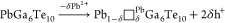
1
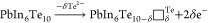
2
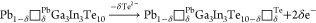
3

The |*S*| values for the majority of samples decrease with the temperature up to the phase transition, in good agreement with the intrinsic decrease of the electrical resistivity described later. Structural transition causes steplike changes in *S*(*T*) for all investigated samples or even changes from n- to p-type conductivity for samples with *x* = 2–4.

The changes in the Seebeck coefficient according to the composition in the Pb_*y*_Ga_6–*x*_In_*x*_Te_10_ samples are represented in Figure 6b. The very high positive values of *S* for the sample with *x* = 0 suggest a very low carrier concentration of holes in this material (eq 1). Further, the addition of In causes growth of the electron concentration, which is reflected in the p–n transition for α-Pb_*y*_Ga_6–*x*_In_*x*_Te_10_ with 1 ≤ *x* ≤ 2 (eqs 2 and 3).

**Figure 7:**
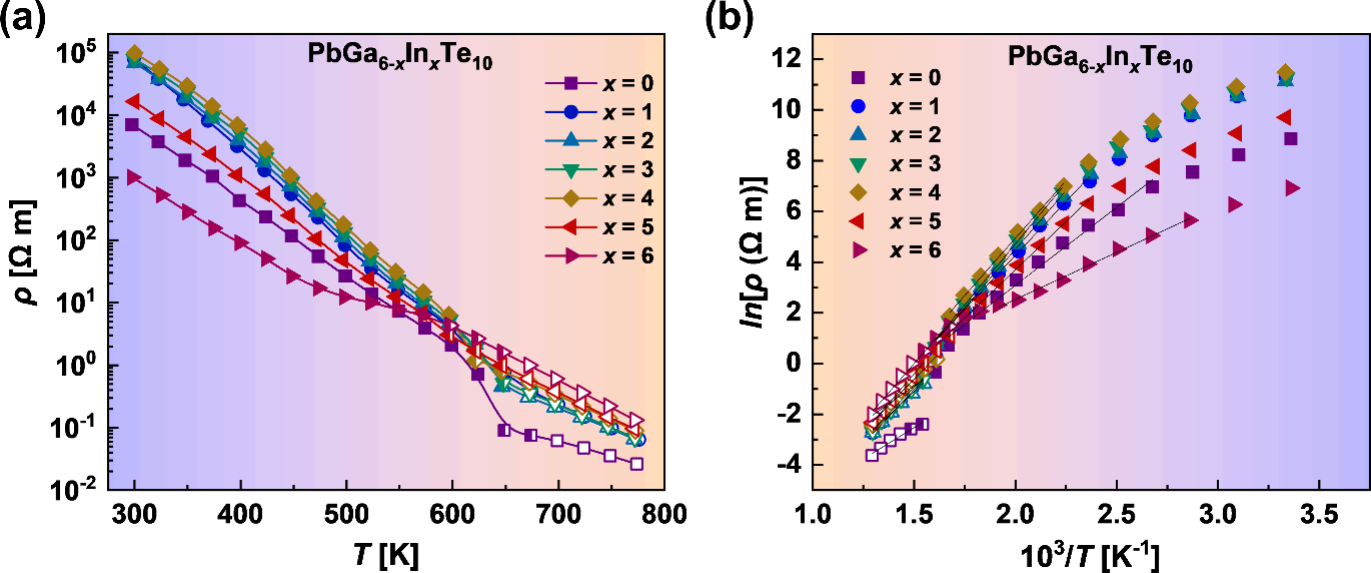
Electrical resistivity (a) and Arrhenius plot (b) of the electrical resistivity for the Pb_*y*_Ga_6–*x*_In_*x*_Te_10_ samples. Filled symbols correspond to the temperature range of existence of the α modification and open symbols to that of the β modification.

Figure 7 shows the electrical resistivity (a) and Arrhenius plot (b) of the electrical resistivity for Pb_*y*_Ga_6–*x*_In_*x*_Te_10_ samples over the entire temperature range of 298–773 K. As expected for undoped, semiconducting, relatively wide-band-gap materials, the values of ρ are high and decrease with increasing temperature. This observation can also be connected with the low carrier concentration and almost equal amounts of majority and minority carriers, which is in agreement with valence-precise PbGa_6–*x*_In_*x*_Te_10_ starting compositions. It is noteworthy that the measured temperature dependencies of the electrical resistivity correspond well with the behavior of the Seebeck coefficient for the PbGa_6–*x*_In_*x*_Te_10_ samples.

The electrical resistivity for Pb_*y*_Ga_6–*x*_In_*x*_Te_10_ increases with increasing *x*, and the sample with *x* = 4 shows the highest ρ among the investigated samples over the entire temperature range (Figure 7a). Such behavior can be explained by the above-proposed defect formation mechanism (eqs 1–3). Particularly, the rise of the electron concentration with In substitution in the PbGa_6–*x*_In_*x*_Te_10_ samples compensates for the existing holes, leading to the rise of the electrical resistivity. With a further increase of the In content in the Pb_*y*_Ga_6–*x*_In_*x*_Te_10_ samples, the resistivity decreases again, and the sample with *x* = 6 shows the weakest decreasing temperature dependence. Characteristic inflections on the ρ(*T*) curves correspond to the polymorphic phase transitions and were also reported for other members of the filled β-Mn-type phases.^22^ The high electrical resistivity of the Pb_*y*_Ga_6–*x*_In_*x*_Te_10_ samples is in good agreement with the very low values of the charge-carrier concentration (*n*_H_ ≈ 10^13^–10^14^ cm^–3^); however, the Hall measurements were too noisy to determine more precisely the values of *n*_H_.

The electrical conductivity activation energies, estimated from the Arrhenius plot of the electrical resistivity (Figure 7b) for the Pb_*y*_Ga_6–*x*_In_*x*_Te_10_ samples, are given in Table 2. Analyzing the Arrhenius plot of the electrical resistivity, we can distinguish at least three regions of changing slopes. A high-temperature region can be described as the intrinsic semiconductor behavior with slightly different slopes before and after the structural transition for a majority of the samples. The values of *E*_a_ before the phase transition for the α-Pb_*y*_Ga_6–*x*_In_*x*_Te_10_ samples are growing with increasing *x* from 1.06(4) to 1.65(5) eV for samples with *x* = 0 and 3, respectively. With a further increase of *x*, the values of *E*_a_ are decreasing to 0.64(2) eV for sample with *x* = 6. The obtained values of the activation energies roughly agree with the literature data of the optical band gaps of the end members of the investigated solid solutions (Table 2). For the case of PbIn_6_Te_10_, the lower band gap and higher contribution of In partial states to the density of states (DOS) were reported by Reshak et al.^40^ The authors also showed p-type conduction for the ideal β-PbIn_6_Te_10_. However, the n-type conduction achieved in this work is due to the Te vacancies that occurred in the real system.

**Table 2:** Activation Energies *E*_a_ for Pb_*y*_Ga_6–*x*_In_*x*_Te_10_ Samples (α-Phase)

*x*	*E*_a_ [eV]	*E*_g_ [eV] (literature)
0	1.06(4)	1.35(1)^41^
1	1.46(2)	
2	1.59(3)	
3	1.65(5)	
4	1.56(4)	
5	1.31(3)	
6	0.64(2)	1.08^42^

**Figure 8:**
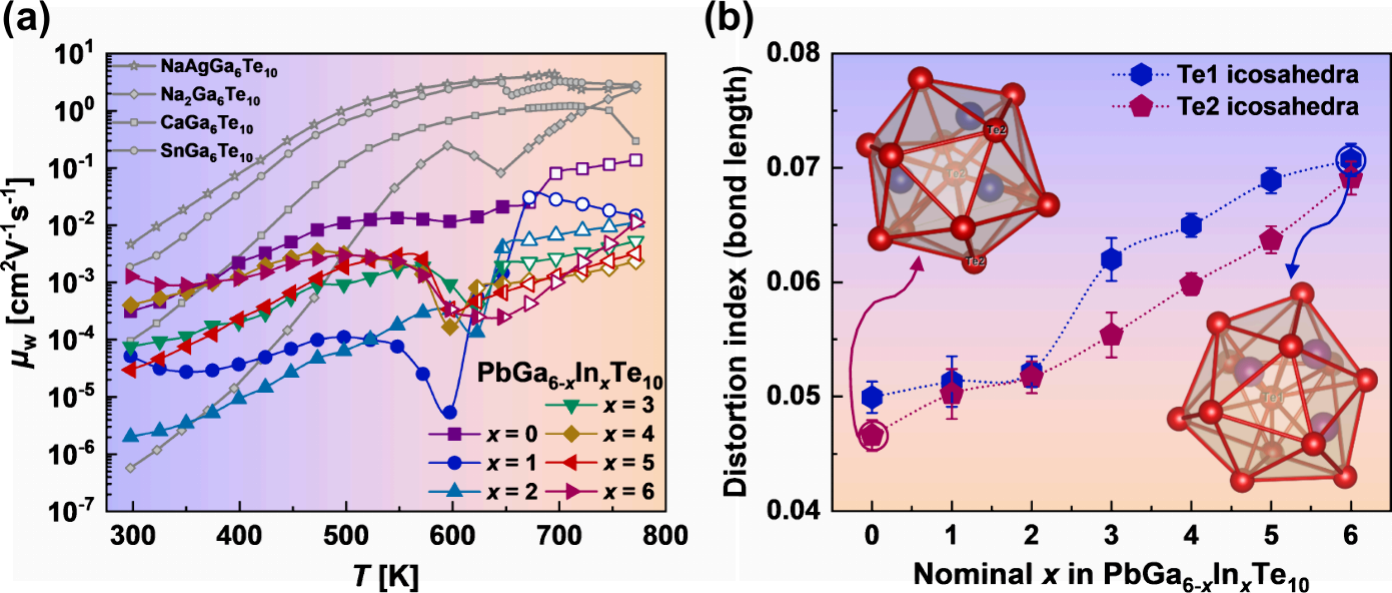
Weighted mobility (a) of filled β-Mn-type phases as a function of the temperature. Filled symbols correspond to the temperature range of existence of the α modification and open symbols to that of the β modification. Distortion index (b) of Te icosahedra determined by Baur’s method^38^ as a function of *x* in Pb_*y*_Ga_6–*x*_In_*x*_Te_10_.

In order to better understand the electronic transport properties in the Pb_*y*_Ga_6–*x*_In_*x*_Te_10_ samples, we calculated the weighted mobility (electron mobility weighted by the density of electronic states) based on the electrical resistivity and Seebeck coefficient measurements, using the equation proposed by Snyder et al.^43^ For materials with a low mobility of charge carriers, the weighted mobility appears to be a much better measure of the drift mobility than measurements of the Hall mobility.^43^ The results of the estimated weighted mobility of Pb_*y*_Ga_6–*x*_In_*x*_Te_10_ compounds in comparison to other previously investigated *M*Ga_6_Te_10_ materials^22^ are shown in Figure 8a.

As was described above, the Te atoms in the crystal structure of Pb_*y*_Ga_6–*x*_In_*x*_Te_10_ materials can be represented as a packing of [(Ga,In)_3_Te_13_] icosahedra with a Te central atom (Figure 5b). The Ga (In) atoms are located in tetrahedral voids, whereas the Pb atoms occupy half of the distorted octahedral voids in the crystal structure. Calculations of the electronic structure of filled β-Mn-type phases reveal that Te states dominate in the DOS near the Fermi level,^22,40,44^ and this suggests that charge transport goes through the framework built up by the Te atoms. Based on the statement suggested by Slack,^45^ good electronic transport requires high crystal symmetry of the atomic framework. However, the Te framework in filled β-Mn-type phases is highly distorted, with significant variation in the interatomic distances between neighboring Te atoms (Figure 5b). This fact may cause such low values of the weighted mobility and strong scattering of charge carriers. With the substitution of Ga by In in Pb_*y*_Ga_6–*x*_In_*x*_Te_10_, we observed a decrease in the weighed mobility for the majority of samples. To understand a possible structural reason for such a change, we defined the distortion index of Te icosahedra (which form the framework in filled β-Mn-type phases) by Baur’s method^46^ using the software *Vesta*(47) and crystallographic information obtained from Rietveld refinement. Distortion indexes were defined by use of the equation^46^
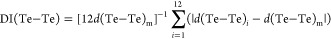
where *d*(Te–Te)_*i*_ stands for the individual distances from the central Te atom to the Te atoms in the corners of icosahedra and m signifies the mean value for each icosahedron. Interestingly, with an increase of *x* in Pb_*y*_Ga_6–*x*_In_*x*_Te_10_, we noticed a growth of the distortion index (Figure 8b). Moreover, such a growth of framework distortions introduces additional strain field fluctuation, which is discussed in the following section.

### 3.4 Thermal Transport

**Figure 9:**
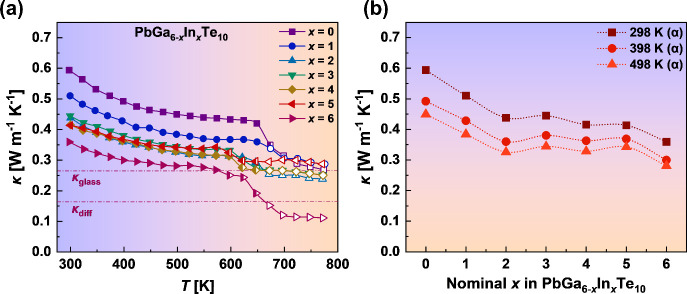
Total thermal conductivity of Pb_*y*_Ga_6–*x*_In_*x*_Te_10_ samples as a function of the temperature (a) and as a function of *x* at selected temperatures (b). Filled symbols correspond to the temperature range of existence of the α modification and open symbols to that of the β modification. κ_glass_ indicates the glass limit and κ_diff_ the diffuson limit of the theoretical minimum lattice thermal conductivity of PbIn_6_Te_10_.

Figure 9a shows the total thermal conductivity (κ) of the studied Pb_*y*_Ga_6–*x*_In_*x*_Te_10_ samples after sintering with >98% of theoretical density. All specimens possess extremely low thermal conductivity in the whole measured temperature range, decreasing from 0.36–0.59 W m^–1^ K^–1^ at 298 K to 0.11–0.29 W m^–1^ K^–1^ at 773 K, which are among the lowest values observed in crystalline materials. We want to point out that a drop in the thermal conductivity in the region of the polymorphic phase transitions between 580 and 700 K is caused, most probably, by the latent heat of the phase transitions, which is not considered in the Dulong–Petit formulation of the heat capacity.^48^ Moreover, the observed changes in the microstructure during the phase transition (Figure S7) can also affect the thermal conductivity. In our case, the total κ is mainly contributed by the lattice thermal conductivity (κ_L_) due to the very high electrical resistivity of the investigated samples.

Due to the change of the dominating defect mechanism, which causes the self-compensation of charge carriers, a change from p- to n-type conductivity was observed for Pb_*y*_Ga_6–*x*_In_*x*_Te_10_ materials with increasing *x*. Such an effect also leads to a decrease in the *ZT* parameters due to the presence of two types of charge carriers and later to a growth of *ZT* for the samples with the highest In content (Figure S11). Nevertheless, due to the relatively wide band gaps in the Pb_*y*_Ga_6–*x*_In_*x*_Te_10_ materials, the carrier concentration is very low, which leads to a low TE performance. Therefore, the increase of the carrier concentration due to the introduction of intrinsic defects or extrinsic doping should improve the overall TE performance of the studied materials.

In this work, we want to understand how the nature of atoms that occupy the tetrahedral voids influences the thermal transport of filled β-Mn-type phases. To address this challenge, we measured the ultrasonic properties and combined the obtained results with the crystal structure features and lattice thermal conductivity data. The evaluation of the main mechanisms responsible for the phonon transport in Pb_*y*_Ga_6–*x*_In_*x*_Te_10_ materials was carried out using the Klemens–Callaway calculations.

The main elastic parameters, i.e., Debye temperature Θ_D_, bulk modulus *B*, Young modulus *E*, Poisson ratio ν, Grüneisen parameter γ, and phonon mean free path *l*_ph_, were estimated using the measured longitudinal *v*_l_ and transverse *v*_t_ sound velocity. The data were also used for the estimation of the minimum thermal conductivity κ_glass_ and κ_diff_ of Pb_*y*_Ga_6–*x*_In_*x*_Te_10_ materials (Table 3).

**Table 3:** Elastic and Thermal Transport Properties of the Pb_*y*_Ga_6–*x*_In_*x*_Te_10_ Samples

*x*	*v*_l_ [m s^–1^]	*v*_t_ [m s^–1^]	*v*_m_ [m s^–1^]	Θ_D_ [K]	*B* [GPa]	*E* [GPa]	ν	γ	*l*_ph_ [Å]	κ_glass_[W m^–1^K^–1^]	κ_diff_[W m^–1^K^–1^]
0	2659	1606	1775	167.2	21.4	36.8	0.213	1.34	7.8	0.33	0.21
1	2623	1558	1725	161.8	21.6	35.4	0.227	1.40	7.1	0.32	0.20
2	2557	1513	1676	155.7	20.6	33.3	0.231	1.41	6.4	0.30	0.19
3	2511	1485	1645	152.0	20.0	32.3	0.231	1.41	6.8	0.29	0.19
4	2461	1449	1606	147.8	19.5	31.1	0.234	1.43	6.6	0.28	0.18
5	2452	1435	1591	145.4	19.6	30.7	0.240	1.45	6.8	0.28	0.18
6	2341	1410	1560	141.4	16.9	29.0	0.215	1.35	6.2	0.27	0.17

The procedure for the determination of the elastic and thermal transport characteristics can be found in eqs S1–S11.

Generally, the average speed of sound *v*_m_ decreases from 1775 to 1560 m s^–1^ in Pb_*y*_Ga_6–*x*_In_*x*_Te_10_ samples with increasing In content. Such low values of the speed of sound can be explained by the large unit cell of Pb_*y*_Ga_6–*x*_In_*x*_Te_10_ with heavy constituent atoms. The large unit cell corresponds to the shrinking Brillouin zone, which leads to a reduction of the fraction of acoustic phonons, which are the main contributors to the lattice thermal conductivity.^49^ For Pb{Ga,In}_6_Te_10_ compounds, Cheng et al.^50^ showed an extremely low cutoff frequency of the acoustic branch of ∼0.5 THz, which is one of the lowest values in inorganic materials.^50^ Consequently, in our case, the synergistic effect of the large number of atoms in the primitive cell (*N* = 102) of Pb_*y*_Ga_6–*x*_In_*x*_Te_10_ (i.e., small Brillouin zone) and the weakly bonded Pb^2+^ ions^16^ is mainly responsible for the low speed of sound and significantly restricted phonon transport. Furthermore, the bulk (*B*) and elastic (*E*) moduli, calculated from the ultrasonic measurements, are lower than those in the case of typical TEs,^49,51,52^ showing that the low bond stiffness is responsible for the observed low lattice thermal conductivity.^53^

Although we do not observe any clear dependence of the Grüneisen parameters on *x*, the estimated Grüneisen parameters γ ∼ 1.34–1.45 for the Pb_*y*_Ga_6–*x*_In_*x*_Te_10_ materials with close-packed tetrahedra structure are significantly higher in comparison to the diamond-like semiconductors with tetrahedral coordination, which typically show γ ∼ 0.5–0.7.^8^ The Grüneisen parameter (γ) is widely employed to evaluate the measure of lattice anharmonicity, which is defined as the change of the phonon frequency as a function of volume.^49^ In general, materials with large atomic coordination numbers, rattling atoms, or bonding instabilities with lone-pair electrons are expected to have large anharmonicity of lattice vibrations.^8,15,54,55^ As we recently reported, the presence of lone-pair-like interactions on Te atoms in filled β-Mn-type phases causes distortions in bond distances and angles; this fact may be the most realistic reason for significant Grüneisen parameters and lattice anharmonicity in Pb_*y*_Ga_6–*x*_In_*x*_Te_10_ materials. In turn, the alteration in the bond lengths and bond angles in the crystal lattice is reflected in the local bulk moduli. This, in addition to mass field fluctuations, can increase the strain field and hence intensify point defect scattering.^8^

For a more detailed understanding of heat transport, we have also calculated the “minimum thermal conductivity” by means of two different approaches. The lattice thermal conductivity of all samples in the range of the low-temperature α modification approaches the glass limit for the thermal conductivity κ_glass_ based on the maximum phonon scattering approach according to Cahill’s formulation.^56^ However, at elevated temperatures, in the range of the existence of disordered β modification, all samples cross the κ_glass_ limit; therefore, this theory cannot describe such behavior. Consequently, the diffuson-based model adapted to disordered systems was used to estimate a minimum of the thermal conductivity κ_diff_.^57^ Most of the samples studied approach κ_diff_ at elevated temperatures; however, PbIn_6_Te_10_ breaks even this minimal threshold, confounding the understanding of heat transport in this material using classical phonon-transport theories.

**Figure 10:**
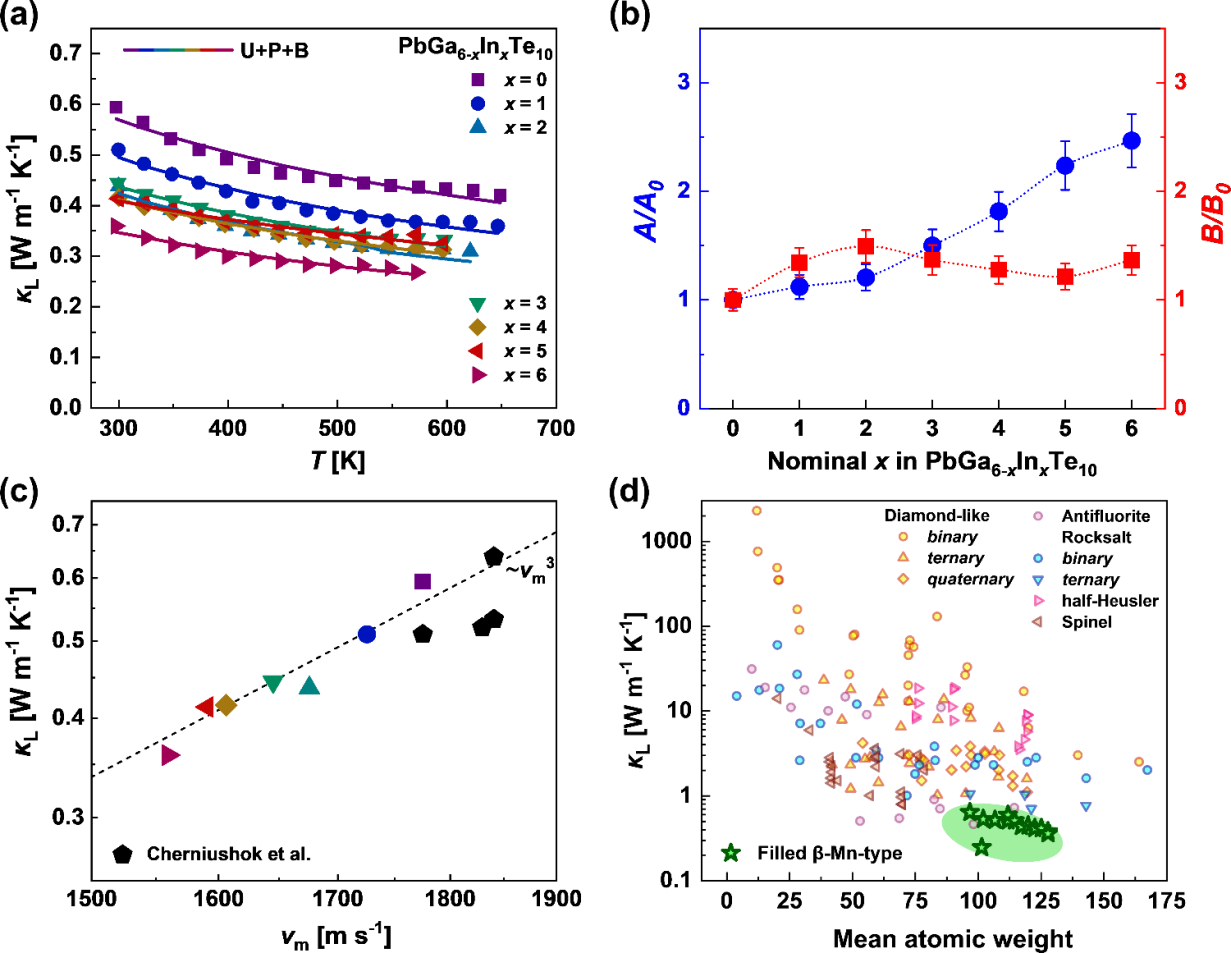
(a) Lattice thermal conductivity of the Pb_*y*_Ga_6–*x*_In_*x*_Te_10_ samples before the phase transition. Solid lines were calculated using the Klemens–Callaway approach accounting for Umklapp scattering (U), point defect scattering (P), and grain boundary scattering (B). (b) Phonon scattering mechanisms. The model parameters *A* and *B* define the intensity of phonon scattering on point defects and the three-phonon Umklapp scattering processes, respectively. (c) Experimental lattice thermal conductivity κ_L_ of filled β-Mn-type materials versus mean sound velocity. Data were taken from ref (22) for comparison. (d) Dependence between the mean atomic weight and lattice thermal conductivity at 298 K for the investigated filled β-Mn-type materials in comparison with other families of semiconductors.^52,59−61^

Due to the low electrical conductivity of the investigated materials, the electronic part of the thermal conductivity can be neglected, and we assumed that only the lattice contribution κ_L_ determines the total thermal conductivity (Figure 10a). To further analyze the mechanisms that affect the lattice thermal conductivity for the investigated materials, we used the Klemens–Callaway approach:^58^
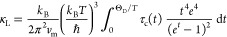
1a

In this model, the main parameters that modify the phonon relaxation time (τ_c_) are related to the phonon–phonon Umklapp scattering, point defect scattering, and grain boundary scattering (eqs S12–S14).

All determined κ_L_(*T*) dependences in the temperature region of the existence of α polymorphs were reasonably well fitted by the Klemens–Callaway model (Figure 10a). The effect of the grain boundaries can be assumed to be roughly the same for all studied samples due to the identical preparation procedure; consequently, our analysis was concentrated on the fitting parameters *A* and *B* (Figure 10b), which reflect the magnitude of phonon scattering on point defects and phonon–phonon Umklapp scattering processes, respectively. The *A* parameter for Pb_*y*_Ga_6–*x*_In_*x*_Te_10_ samples (Figure 10b) increases with increasing In content, suggesting the growth of point defect scattering in the series. This effect may be understood in terms of crystal chemistry because the observed growth of the Pb–Te interatomic distance (Figure S8) in the Pb_*y*_Ga_6–*x*_In_*x*_Te_10_ series should lead to bond weakening. On the other hand, as described above, the growth of distortion in the Te framework with the increase of the In content in Pb_*y*_Ga_6–*x*_In_*x*_Te_10_ samples may also be responsible for the increase of point defects in the system.^62^ Point defect scattering in solid solutions is the result of both mass and strain field fluctuations. The calculated scattering parameters following the Abeles model^63^ for the Pb_*y*_Ga_6–*x*_In_*x*_Te_10_ samples are shown in Table S4. The details of the calculations can be found in the Supporting Information. Considering the estimated Γ_m_ values (Table S4), the contribution of mass fluctuation (Ga/In) has only a minor influence on the phonon scattering in the Pb_*y*_Ga_6–*x*_In_*x*_Te_10_ materials. In turn, the Γ_s_ values show that scattering on the strain field dominates. This observation agrees well with the distortion of the Te framework caused by In substitution. The parameter *B* was found to be comparable for all Pb_*y*_Ga_6–*x*_In_*x*_Te_10_ samples, suggesting a comparable effect of the Umklapp phonon–phonon scattering for all representatives. It should be noted that a satisfactory fit to the experimental results was obtained by including only three phonon scattering mechanisms, namely, point defects, Umklapp, and grain boundaries of κ_L_(*T*) for all Pb_*y*_Ga_6–*x*_In_*x*_Te_10_ samples. Nevertheless, it is conceivable that other phonon scattering effects, such as phonon scattering by structural disorder^64^ or phonon resonance scattering^65^), may occur, although their impact is relatively minor in comparison to the aforementioned three phenomena. Furthermore, the average speed of sound correlates well with the lattice thermal conductivities (Figure 10c), and κ_L_ roughly follows the theoretical ∼*v*_m_^3^ dependence^8^ for the investigated filled β-Mn-type materials.

The temperature dependence of the lattice thermal conductivity below 450 K shows a decreasing tendency and is proportional to ∼*T*^–1^, suggesting domination of Umklapp scattering, and can be explained by the classical phonon–gas theory of thermal conductivity. However, above this temperature, up to the polymorphic phase transition, κ_L_(*T*) becomes almost temperature-independent and may suggest that the diffuson channel dominates the thermal transport in this region.^66,67^

As mentioned above, the presence of the stereochemically active lone-pair-like bonds on Te in the Ga (In)–Te framework induces lattice anharmonicity,^20^ which is reflected in the high Grüneisen parameters. For these reasons, the close-packed tetrahedra structures, in particular, filled β-Mn-type materials, show extremely low lattice thermal conductivity with a weak dependence on the mean atomic weight of the constituent elements^22,49^ (Figure 10d). Moreover, the large variety of Te–Pb interactions within the octahedron, i.e., the large spectrum between *d*(Pb–Te)_min_ and *d*(Pb–Te)_max_, indicates the presence of bonding inhomogeneity, leading to the troublesome phonon transport. In the particular case of Pb_*y*_Ga_6–*x*_In_*x*_Te_10_ materials, the observed decrease in the lattice thermal conductivity with *x* is caused by the expansion of the [(Ga,In)_3_Te_13_] icosahedra and the simultaneous growth of the Pb–Te interatomic distance as follows from the analysis of the crystal structure and elastic properties. The increase of the Pb–Te distance means an effective reduction of the bonding energy, which allows the intensification of the thermal vibrations. These result in the growth of point defects, as was confirmed by the Klemens–Callaway analysis.

## 4 Conclusions

In this work, the influence of cation substitution in tetrahedral voids on the crystal-chemical, thermal, and TE properties of the filled β-Mn-type materials with the composition Pb_*y*_Ga_6–*x*_In_*x*_Te_10_ was systematically investigated, aiming to explore their potential functionality in thermoelectricity. Structural transitions in the range between 580 and 700 K were discovered here for all studied compositions. The crystal structure model for the new α-PbIn_6_Te_10_ modification with the space group *P*3_2_21 was proposed. Refined lattice parameters show a linear dependence of cell volumes with increasing *x* in PbGa_6–*x*_In_*x*_Te_10_ in agreement with Vegard’s rule.

The Pb_*y*_Ga_6–*x*_In_*x*_Te_10_ materials show very high values of electrical resistivity decreasing with the temperature and change from p- to n-type conductivity. All investigated Pb_*y*_Ga_6–*x*_In_*x*_Te_10_ materials possess extremely low lattice thermal conductivities due to the high anharmonicity of lattice vibrations reflected in the high Grüneisen parameter. κ_L_ decreases with increasing *x* from 0.59 to 0.36 W m^–1^ K^–1^ at 298 K due to the expansion of the [(Ga,In)_3_Te_13_] icosahedra and simultaneous growth of the Pb–Te interatomic distance. Increasing the Pb–Te spacing increases the amplitude of anharmonic lattice vibrations. This leads to a growth of point defects, as derived from Klemens–Callaway’s analysis. This work shows the filled β-Mn-type materials with close-packed tetrahedra structure and composition Pb_*y*_Ga_6–*x*_In_*x*_Te_10_ as a family of materials with extremely low lattice thermal conductivity.

## Supporting Information Available

Powder XRD data, microstructure data, crystallographic information, dependence between the Seebeck coefficient and electrical conductivity, *ZT* parameters, and details of calculations of the elastic and thermal transport properties (PDF)
