# Supplementary material for: Controlled Phonon Transport via Chemical Bond Stretching and Defect Engineering: The Case Study of Filled β-Mn-Type Phases
Source: Inorg Chem. 2024 Sep 13;63(39):18030–42. doi: 10.1021/acs.inorgchem.4c02562 (PMC11445726; doi:10.1021/acs.inorgchem.4c02562)
Supplement: Supplementary file 1 — ic4c02562_si_001.pdf [file ic4c02562_si_001.pdf]

Supporting Information

Controlled phonon transport via chemical bond stretching and defect engineering: the case study of filled β-Mn-type phases

Oleksandr Cherniushok[1]*, Taras Parashchuk[1], Raul Cardoso-Gil[2], Yuri Grin[2]*, and Krzysztof T. Wojciechowski[1]*

[1]Thermoelectric Research Laboratory, Department of Inorganic Chemistry, Faculty of Materials Science and Ceramics, AGH University of Krakow, Mickiewicza Ave. 30, 30-059 Krakow, Poland

[2]Max-Planck-Institut für Chemische Physik fester Stoffe, Nöthnitzer Str. 40, 01187 Dresden, Germany

*E-Mails: sashach@agh.edu.pl; grin@cpfs.mpg.de; wojciech@agh.edu.pl

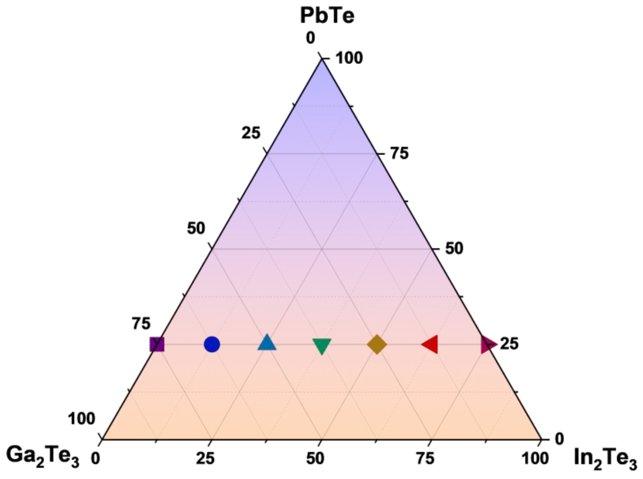

Figure S1. Composition of studied samples in the system PbTe-Ga_2Te_3-In_2Te_3.

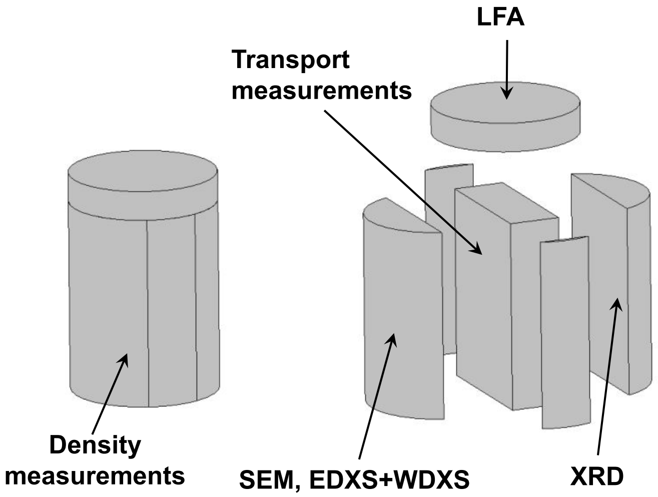

Figure S2. Preparation of samples for the characterization of physical properties.

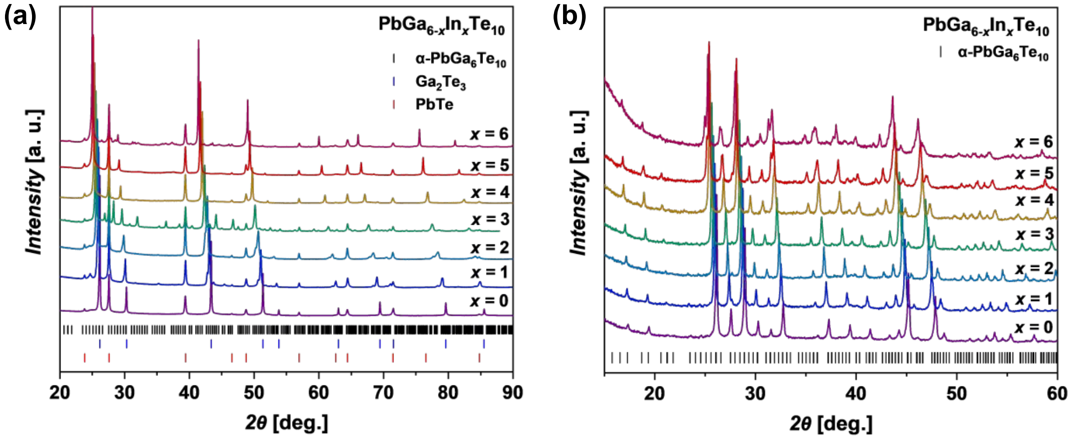

Figure S3. Powder XRD patterns of $Pb_yGa_{6-x}In_xTe_{10}$ samples after DSC measurement above melting temperature (a) and sintered pellet samples after measurements of thermoelectric properties (b). After the DSC measurements above the melting temperature, the XRD reflections belong to PbTe and $(Ga,In)_2Te_3$ indicating the decomposition of samples after melting, while after SPS at 773 K and measurements of thermoelectric properties, the XRD patterns can be indexed in $P3_221$ space group suggesting the existence of the α-modification of the $Pb_yGa_{6-x}In_xTe_{10}$ compounds.

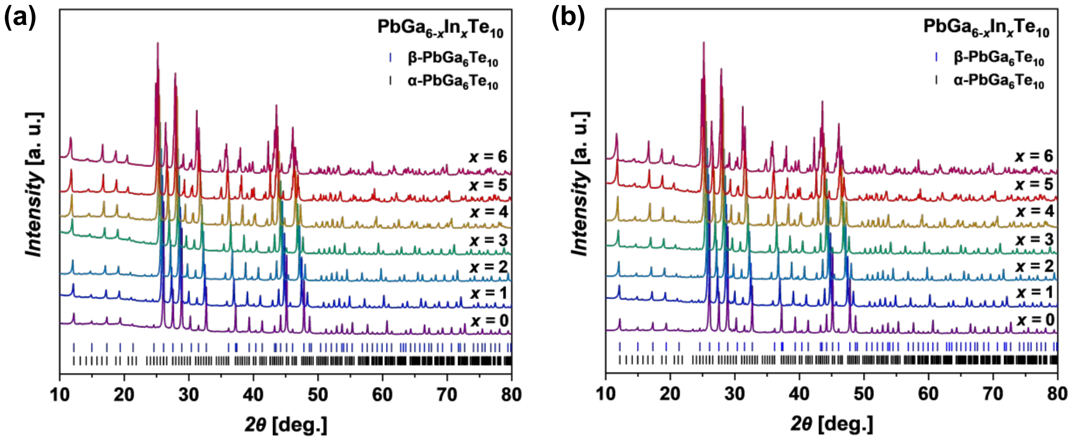

Figure S4. Powder XRD patterns of $PbGa_{6-x}In_xTe_{10}$ powdered samples after SPS (a) and after measurements of thermoelectric properties (b).

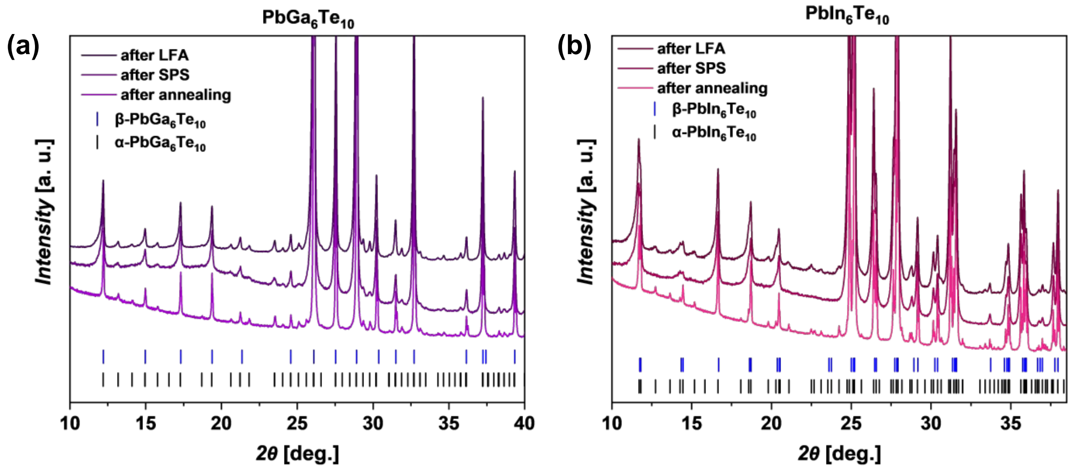

Figure S5. Powder XRD patterns of $PbGa_6Te_{10}$ (a) and $PbIn_6Te_{10}$ (b) samples with magnified *2θ* region. The superstructure reflections of the α-modification of filled β-Mn type compounds are less intensive for $PbIn_6Te_{10}$ than for $PbGa_6Te_{10}$ in agreement with the lower temperature of the phase transition from α- (space group $P3_221$) to β-phase (space group *R*32). Also, after SPS, the superstructure reflections of the α-modification are very weak or even absent indicating the dominative existence of high-temperature disordered β-modification.

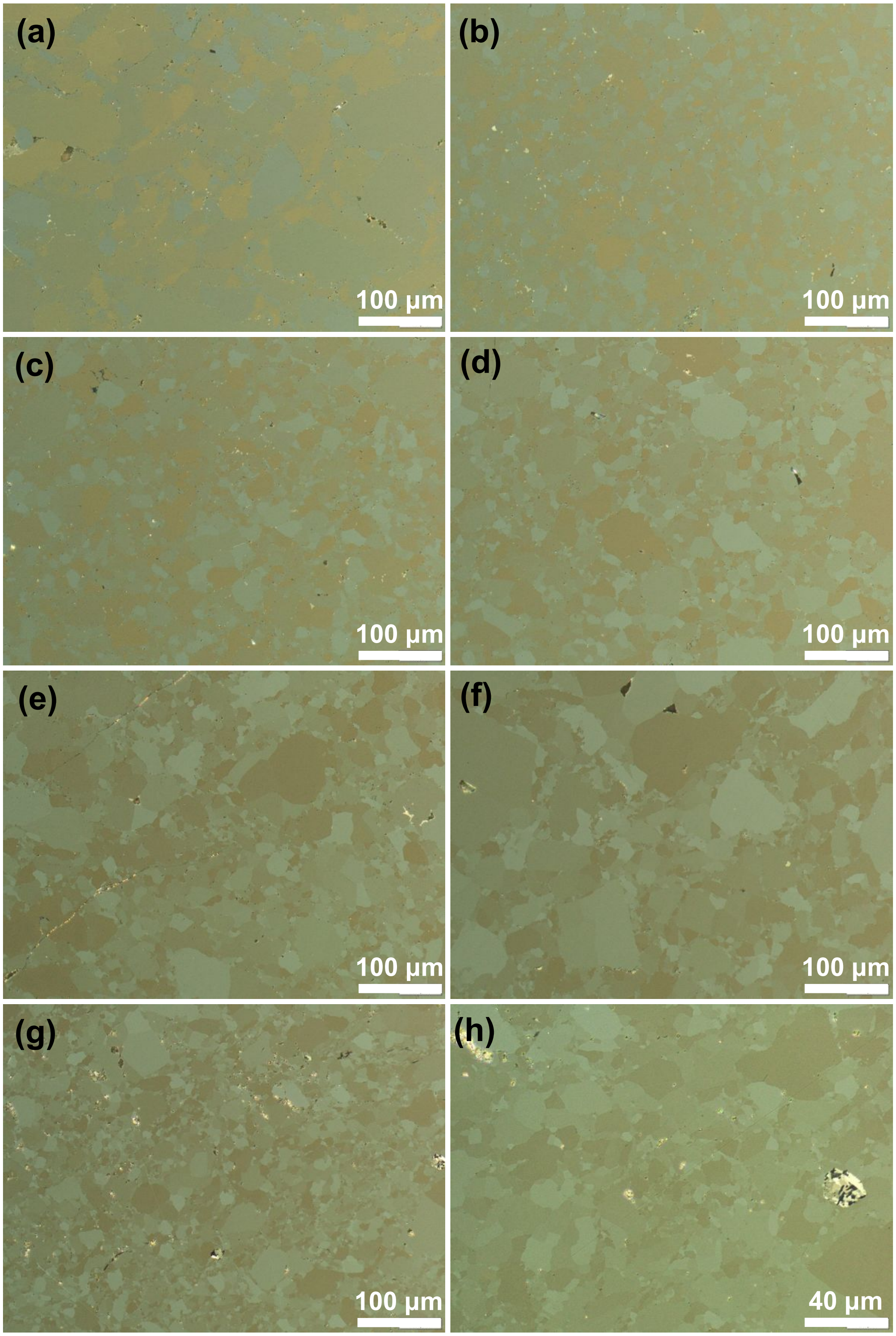

Figure S6. Optical polarized-light micrographs of α-$Pb_yGa_{6-x}In_xTe_{10}$ samples after SPS treatment: (a) $x = 0$; (b) $x = 1$; (c) $x = 2$; (d) $x = 3$; (e) $x = 4$; (f) $x = 5$; and (g,h) $x = 6$.

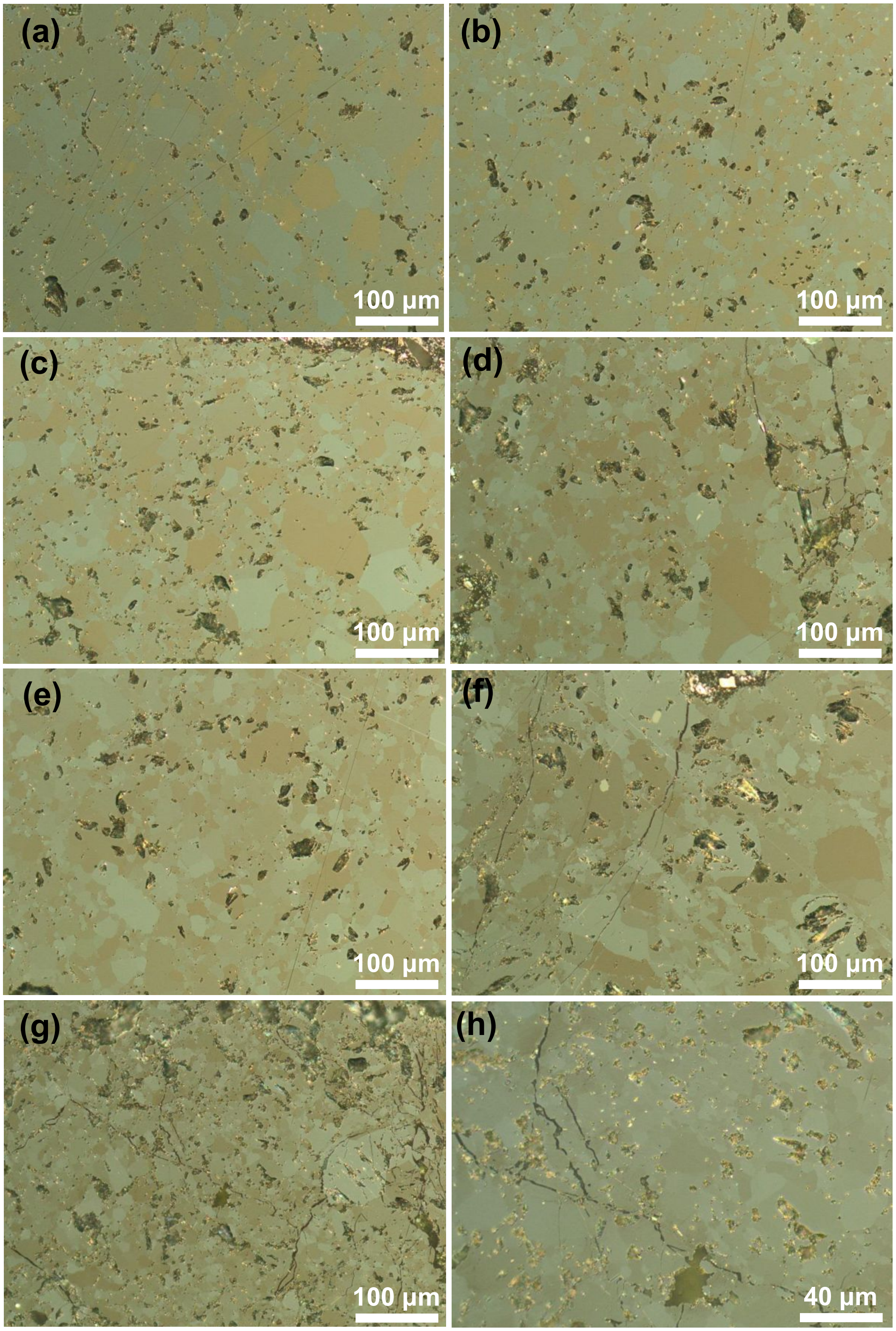

Figure S7. Optical polarized-light micrographs of α-$Pb_yGa_{6-x}In_xTe_{10}$ samples after TE properties measurements up to 773 K: (a) $x = 0$; (b) $x = 1$; (c) $x = 2$; (d) $x = 3$; (e) $x = 4$; (f) $x = 5$; and (g,h) $x = 6$.

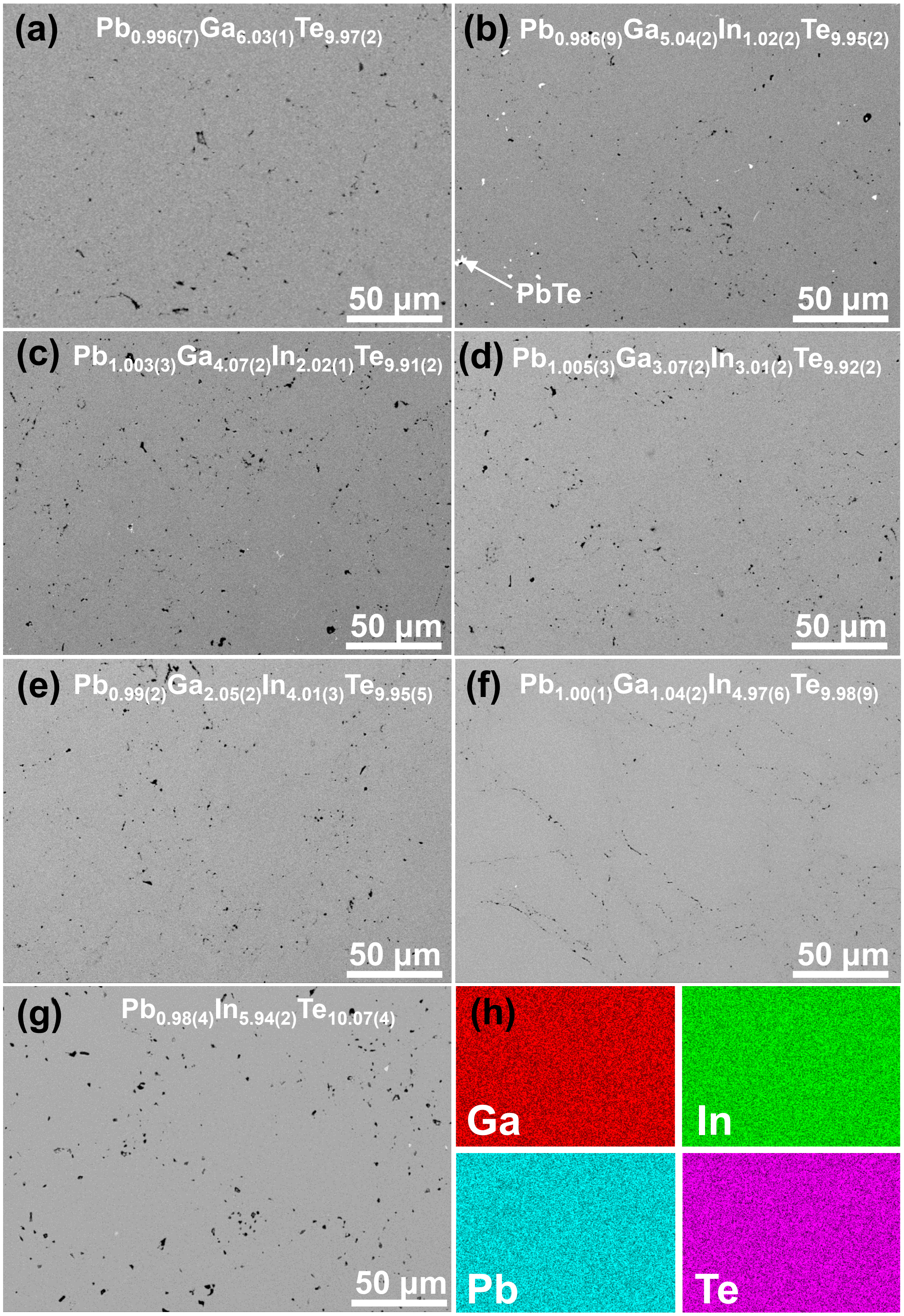

Figure S8. Backscattered electron images of $PbGa_{6-x}In_xTe_{10}$ samples after SPS treatment: (a) $x = 0$; (b) $x = 1$; (c) $x = 2$; (d) $x = 3$; (e) $x = 4$; (f) $x = 5$; and (g) $x = 6$. Chemical composition of phases is shown according to WDXS analysis. (h) EDXS element mapping for $PbGa_{6-x}In_xTe_{10}$ sample with $x = 3$.

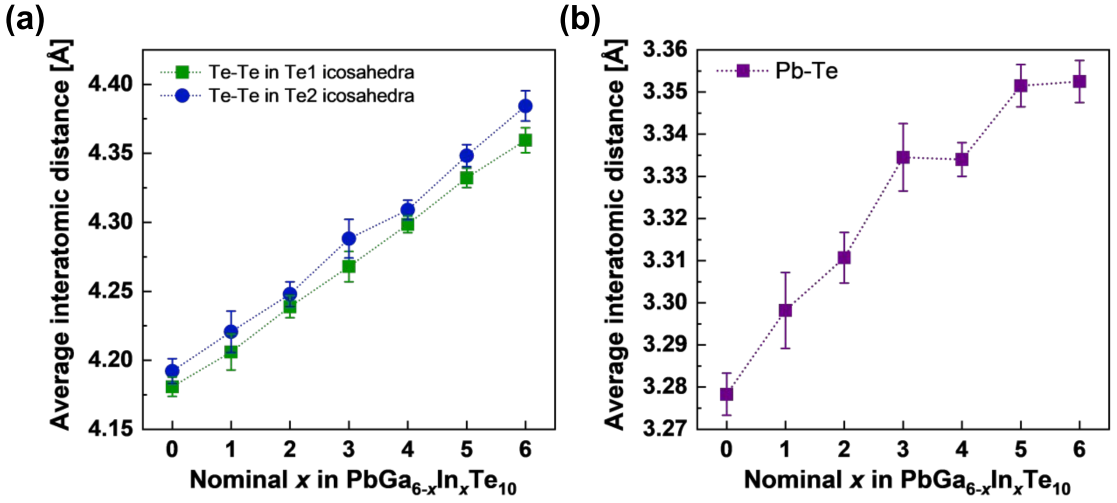

Figure S9. (a) Average Te-Te interatomic distance in Te1 and Te2 icosahedra and (b) Pb-Te interatomic distance as a function of x in $Pb_yGa_{6-x}In_xTe_{10}$ samples determined from Rietveld refinement.

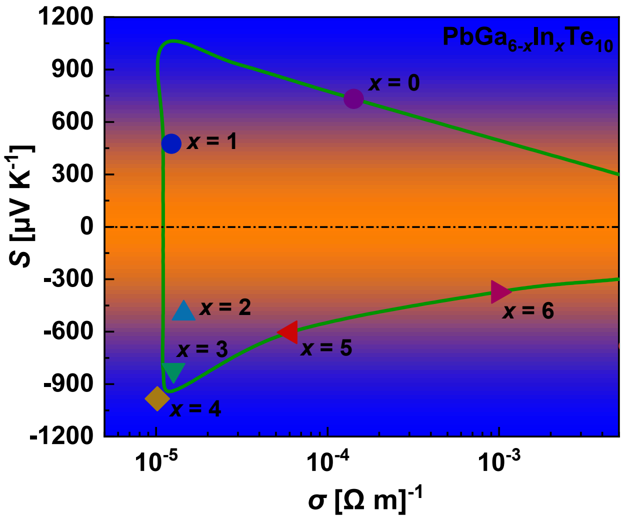

Figure S10. Seebeck coefficient of $PbGa_{6-x}In_xTe_{10}$ samples as a function of electrical conductivity at 298 K. Lines are a guide for the eye.

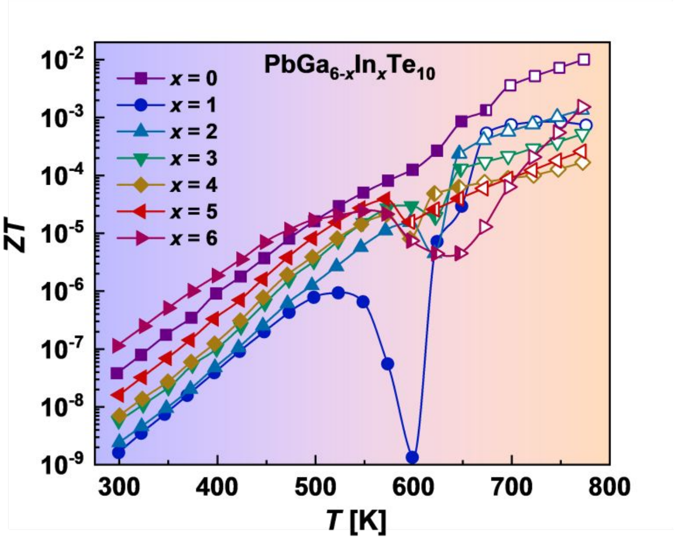

Figure S11. Temperature-dependent dimensionless thermoelectric figure of merit *ZT* for $Pb_yGa_{6-x}In_xTe_{10}$ samples. Filled symbols correspond to the temperature range of existence of α-modification, open symbols to β-modification.

Table S1. Atomic coordinates and isotropic displacement parameters for α-$PbGa_6Te_{10}$

Atom	Site	*x/a*	*y/b*	*z/c*	B_{eq} [$Å^2$]	Occupancy
Pb1	6*c*	0.9201(2)	1.2617(2)	0.1587(2)	1.98(2)	1
Ga1	6*c*	0.2487(4)	0.5743(5)	-0.0843(4)	1.43(11)	1
Ga2	6*c*	0.4788(4)	0.8841(4)	-0.0629(4)	0.97(11)	1
Ga3	6*c*	0.4114(4)	0.6596(4)	0.0779(4)	1.08(9)	1
Ga4	6*c*	0.7820(4)	0.9243(4)	-0.0707(4)	0.54(12)	1
Ga5	6*c*	0.1865(4)	0.7446(4)	0.0756(3)	0.32(10)	1
Ga6	6*c*	0.9156(4)	0.9274(4)	-0.2523(3)	1.46(8)	1
Te1	6*c*	0.1974(3)	0.5661(3)	0.0623(2)	0.85(3)	1
Te2	6*c*	0.9651(3)	1.1020(2)	-0.0618(2)	1.12(6)	1
Te3	6*c*	0.4826(3)	0.9450(3)	-0.2082(2)	1.25(6)	1
Te4	6*c*	0.6709(2)	1.0034(3)	-0.0019(1)	0.68(4)	1
Te5	6*c*	0.4665(3)	0.6982(3)	-0.0625(2)	1.09(6)	1
Te6	3*b*	0.8431(3)	0	1/6	1.08(9)	1
Te7	6*c*	0.7190(3)	0.8722(2)	-0.2112(2)	1.20(7)	1
Te8	6*c*	0.2119(2)	0.3901(3)	-0.1199(2)	1.25(7)	1
Te9	3*a*	0.7519(3)	0.7519(3)	0	1.03(10)	1
Te10	6*c*	0.3421(2)	0.9176(2)	0.0092(2)	1.21(4)	1
Te11	6*c*	0.1675(2)	0.6619(2)	-0.1708(3)	1.46(6)	1

Table S2. Atomic coordinates and isotropic displacement parameters for α-$PbIn_6Te_{10}$

Atom	Site	*x/a*	*y/b*	*z/c*	B_{eq} [Å^2]	Occupancy
Pb1	6*c*	0.9222(1)	1.2635(1)	0.1579(1)	2.08(2)	1
In1	6*c*	0.2423(2)	0.5662(2)	-0.0834(2)	1.65(5)	1
In2	6*c*	0.4767(3)	0.8868(2)	-0.0645(2)	1.01(4)	1
In3	6*c*	0.4117(3)	0.6574(3)	0.0779(2)	1.33(4)	1
In4	6*c*	0.7755(3)	0.9184(3)	-0.0698(3)	1.98(5)	1
In5	6*c*	0.1860(3)	0.7471(3)	0.0732(2)	1.40(5)	1
In6	6*c*	0.9107(3)	0.9221(3)	-0.2503(2)	0.92(4)	1
Te1	6*c*	0.1941(3)	0.5628(3)	0.0624(2)	1.39(4)	1
Te2	6*c*	0.9668(3)	1.1050(3)	-0.0657(2)	1.19(4)	1
Te3	6*c*	0.4792(3)	0.9534(3)	-0.2070(2)	1.76(5)	1
Te4	6*c*	0.6695(3)	1.0007(4)	0.00371(8)	1.12(3)	1
Te5	6*c*	0.4645(2)	0.6939(2)	-0.0664(2)	0.56(3)	1
Te6	3*b*	0.8338(3)	1	1/6	1.66(7)	1
Te7	6*c*	0.7103(3)	0.8589(2)	-0.2090(2)	1.32(4)	1
Te8	6*c*	0.2067(2)	0.3775(3)	-0.1189(2)	2.04(5)	1
Te9	3*a*	0.7371(3)	0.7371(3)	0	1.75(7)	1
Te10	6*c*	0.3359(3)	0.9265(2)	0.0030(2)	2.03(4)	1
Te11	6*c*	0.1614(2)	0.6578(2)	-0.1726(2)	1.25(4)	1

Table S3. Atomic coordinates and isotropic displacement parameters for α-$PbGa_3In_3Te_{10}$

Atom	Site	*x/a*	*y/b*	*z/c*	B_{eq} [Å^2]	Occupancy
Pb	6*c*	0.9212(4)	1.2603(5)	0.1571(3)	1.14(8)	0.877(2)
Ga1	6*c*	0.2475(8)	0.5684(8)	-0.0786(5)	1.1(2)	0.452(4) Ga + 0.548(4) In
Ga2	6*c*	0.4774(9)	0.8869(8)	-0.0670(6)	1.1(3)	0.516(4) Ga + 0.484(4) In
Ga3	6*c*	0.4094(9)	0.6522(9)	0.0863(6)	1.2(3)	0.591(4) Ga + 0.409(4) In
Ga4	6*c*	0.7749(8)	0.9189(8)	-0.0686(6)	1.2(2)	0.343(4) Ga + 0.657(4) In
Ga5	6*c*	0.1898(8)	0.7459(8)	0.0764(5)	1.2(2)	0.317(4) Ga + 0.683(4) In
Ga6	6*c*	0.9200(10)	0.9269(10)	-0.2531(7)	1.4(3)	0.778(4) Ga + 0.222(4) In
Te1	6*c*	0.1949(7)	0.5612(7)	0.0669(4)	1.2(2)	1
Te2	6*c*	0.9690(7)	1.1025(7)	-0.0599(5)	1.3(2)	1
Te3	6*c*	0.4753(7)	0.9462(7)	-0.2052(4)	1.1(2)	1
Te4	6*c*	0.6703(8)	1.0023(10)	0.0042(3)	1.41(12)	1
Te5	6*c*	0.4654(7)	0.6971(7)	-0.0625(4)	1.3(2)	1
Te6	3*b*	0.8431(7)	0	1/6	0.9(3)	1
Te7	6*c*	0.7191(8)	0.8716(7)	-0.2170(4)	1.5(2)	1
Te8	6*c*	0.2112(6)	0.3828(7)	-0.1187(5)	1.2(2)	1

Te9	3*a*	0.7350(8)	0.7350(8)	0	1.4(3)	1
Te10	6*c*	0.3427(5)	0.9272(5)	0.0080(4)	1.2(2)	1
Te11	6*c*	0.1690(6)	0.6604(7)	-0.1725(5)	1.24(15)	1

Elastic properties

The bulk modulus was calculated using the following equation [1]:

$$B=\rho\left(\upsilon_L^2-\frac{4}{3}\upsilon_T^2\right), \tag{S1}$$

where ρ is the material density.

The shear modulus was calculated as:

$$G=\upsilon_T^2\rho. \tag{S2}$$

The Young's modulus is calculated as:

$$E=\frac{9BG}{3B+G}. \tag{S3}$$

The Poisson's ratio is calculated as:

$$\nu=\frac{E-2G}{2G}. \tag{S4}$$

The Debye temperatures were calculated using the following expression [2]:

$$\Theta_D=\frac{h}{k_B}\left[\frac{3n}{4\pi}\left(\frac{N_A\rho}{M}\right)\right]^{1/3}v_m, \tag{S5}$$

where h is Planck's constant, k_B is Boltzmann's constant, N_A is Avogadro's number, M is the molecular weight, n is the number of atoms in the molecule, and v_m is the averaged wave velocity integrated over several crystal directions [2]:

$$v_m = \left[\frac{1}{3}\left(\frac{2}{v_t^3} + \frac{1}{v_l^3}\right)\right]^{-1/3}, \quad \text{(S6)}$$

where v_l and v_t are the longitudinal and transverse sound velocities, respectively. Grüneisen parameters γ were calculated using the following formula [3]:

$$\gamma = \frac{3}{2}\left(\frac{1+\nu}{2-3\nu}\right). \quad \text{(S7)}$$

Thermal transport properties

The phonon mean free paths were calculated by [4]:

$$l_{ph} = \frac{3\kappa_{lat}}{C_V v_m}. \quad \text{(S8)}$$

Considering the ultrasonic data, the lattice thermal conductivity was also calculated as follows [5]:

$$\kappa_L = K\frac{\overline{M}\Theta_D^3\delta}{\gamma^2 n^{\frac{2}{3}} T}. \quad \text{(S9)}$$

Here n is the number of atoms in the primitive unit cell, δ^3 is the volume per atom, Θ_D is the Debye temperature, $\overline{M}$ is the average mass of the atoms in the crystal, and C is a collection of physical constants ($K \approx 3.1\times10^{-6}$ if κ_L is in $Wm^{-1}K^{-1}$, $\overline{M}$ in amu, and δ in Angstroms).

According to Cahill's formulation based on the maximum phonon scattering approach, the glassy limit for the thermal conductivity κ_{glass} was estimated by [6]:

$$\kappa_{glass} = \frac{1}{2}\left(\frac{\pi}{6}\right)^{1/3} k_B V^{-2/3}\left(2v_t + v_l\right), \quad \text{(S10)}$$

where V is the average volume per atom calculated from the refined lattice parameters. The diffusion-based minimum of the thermal conductivity κ_{diff} was calculated using the Cahill plot, as proposed by Agne et al. [7] expression:

$$\kappa_{diff} \approx 0.76 n^{2/3} k_B \frac{1}{3}\left(2v_t + v_l\right) \approx 0.63\kappa_{glass}. \quad \text{(S11)}$$

The phonon relaxation time (τ_c) is calculated using contributions related to point defects scattering (τ_p) [8], phonon-phonon Umklapp scattering (τ_U) [9,10], grain boundary scattering (τ_B), and phonon resonance scattering (τ_R) [11]:

$$\tau_P^{-1} = A\left(\frac{k_B T}{\hbar}\right)^4 t^4, \tag{S12}$$

$$\tau_U^{-1} = B\left(\frac{k_B T}{\hbar}\right)^2 e^{\left(\frac{-\Theta_D}{3T}\right)} T t^2, \tag{S13}$$

$$\tau_B^{-1} = \frac{v_m}{d}, \tag{S14}$$

$$\tau_R^{-1} = \frac{CH\omega^2}{(\omega_0^2-\omega^2)^2+(\Lambda/\pi)^2\omega_0^2\omega^2}, \tag{S15}$$

where, $\hbar = h/(2\pi)$, $t = \hbar\omega/(k_B T)$, A, B, and C are adjustable fitting parameters related to point defect scattering, phonon-phonon Umklapp scattering processes, and resonance phonon scattering, respectively. d is the grain size, H is the half-width of the energy resonance curve ($H = \omega_0\Lambda/2\pi^2$), C is a proportionality factor containing the concentration of oscillators, ω_0 is the resonance circular frequency, and Λ is the logarithmic decrement [$\Lambda = (2Q)^{-1}$].

The ratio of lattice thermal conductivity of the material with point defects κ_L to the material without point defects κ_L^0 can be described as follows [12]:

$$\frac{\kappa_L}{\kappa_L^0} = \frac{tan^{-1}(u)}{u}, u = \left(\frac{\pi^2\Theta_D\Omega}{hv_a^2}\kappa_L^0\Gamma\right)^{1/2} \tag{S16}$$

where u, Ω, v_a, Θ_D, and Γ are the disorder scaling parameter, the average volume per atom, the average sound velocity, the Debye temperature, and the disorder scattering parameter, respectively. The scattering parameter Γ, which indicates the strength of point defect scattering, contains the scattering parameter of mass (Γ_M) and strain field (Γ_S) fluctuations, with an adjustable parameter ε included due to the uncertainty in Γ_S ($\Gamma = \Gamma_M + \varepsilon\Gamma_S$).

Table S4. The room-temperature lattice thermal conductivity (κ_L), disorder scaling parameter (u), scattering parameters (Γ) including mass fluctuation (Γ_M), strain field fluctuation (Γ_S), and the strain field related adjustable parameter ε calculated for $Pb_yGa_{6-x}In_xTe_{10}$ samples.

x	κ_L [$Wm^{-1}K^{-1}$]	u	$\Gamma \times 10^3$	$\Gamma_M \times 10^3$	$\Gamma_S \times 10^3$	ε
0	0.594	-	-	-	-	-
1	0.510	0.909	5.34	0.204	5.14	257
2	0.438	0.958	5.70	0.314	5.39	171
3	0.445	0.953	5.46	0.340	5.12	147
4	0.415	0.974	5.49	0.290	5.20	170
5	0.413	0.976	5.40	0.175	5.22	278
6	0.360	-	-	-	-	-

[1] E.P. Papadakis, E.P. Papdakis, C.A. Stickels, R.C. Innes, An Ultrasonic Technique for Measuring the Elastic Constants of Small Samples, SAE Trans. 104 (1995) 830–837. http://www.jstor.org/stable/44473294.

[2] O.L. Anderson, A simplified method for calculating the debye temperature from elastic constants, J. Phys. Chem. Solids. 24 (1963) 909–917. https://doi.org/10.1016/0022-3697(63)90067-2.

[3] D.S. Sanditov, V.N. Belomestnykh, Relation between the parameters of the elasticity theory and averaged bulk modulus of solids, Tech. Phys. 56 (2011) 1619–1623. https://doi.org/10.1134/S106378421111020X.

[4] H. Xie, S. Hao, S. Cai, T.P. Bailey, C. Uher, C. Wolverton, V.P. Dravid, M.G. Kanatzidis, Ultralow thermal conductivity in diamondoid lattices: high thermoelectric performance in chalcopyrite $Cu_{0.8+y}Ag_{0.2}In_{1-y}Te_2$, Energy Environ. Sci. 13 (2020) 3693–3705. https://doi.org/10.1039/D0EE02323J.

[5] D.T. Morelli, V. Jovovic, J.P. Heremans, Intrinsically minimal thermal conductivity in cubic I-V-VI_2 semiconductors, Phys. Rev. Lett. 101 (2008) 035901. https://doi.org/10.1103/PhysRevLett.101.035901.

[6] D.G. Cahill, R.O. Pohl, Lattice Vibrations and Heat Transport in Crystals and Glasses, Annu. Rev. Phys. Chem. 39 (1988) 93–121. https://doi.org/10.1146/annurev.pc.39.100188.000521.

[7] M.T. Agne, R. Hanus, G.J. Snyder, Minimum thermal conductivity in the context of: Diffuson-mediated thermal transport, Energy Environ. Sci. 11 (2018) 609–616. https://doi.org/10.1039/c7ee03256k.

[8] J. Callaway, Model for lattice thermal conductivity at low temperatures, Phys. Rev. 113 (1959) 1046–1051. https://doi.org/10.1103/PhysRev.113.1046.

[9] C.J. Glassbrenner, G.A. Slack, Thermal Conductivity of Silicon and Germanium from 3°K to the Melting Point, Phys. Rev. 134 (1964) A1058. https://doi.org/10.1103/PhysRev.134.A1058.

[10] G.A. Slack, S. Galginaitis, Thermal Conductivity and Phonon Scattering by Magnetic Impurities in CdTe, Phys. Rev. 133 (1964) A253. https://doi.org/10.1103/PhysRev.133.A253.

[11] R.O. Pohl, Thermal Conductivity and Phonon Resonance Scattering, Phys. Rev. Lett. 8 (1962) 481. https://doi.org/10.1103/PhysRevLett.8.481.
[12] B. Abeles, Lattice thermal conductivity of disordered semiconductor alloys at high temperatures, Phys. Rev. 131 (1963) 1906–1911. https://doi.org/10.1103/PhysRev.131.1906.
